# Gut microbiota-derived imidazole propionate promotes primary sclerosing cholangitis via p38 signalling

**DOI:** 10.1038/s42255-026-01600-1

**Published:** 2026-09-04

**Authors:** Antonio Molinaro, Peder Rustøen Braadland, Guido Carpino, Alba Carreras, Marios Nikolaidis, Petra Hanzely, Katharina R. Beck, Ahmad H. Ali, Lars Bossen, Anna Frank, Annika Lundqvist, Brian D. Juran, Diletta Overi, Lei Geng, Enya Amundsen‑Isaksen, Henrik Mikael Reims, Ida Björk, Krzysztof Grzyb, Andreas Abildgaard, Trine Folseraas, Martin Cornillet, Hólmfríður Helgadóttir, Bryan M. McCauley, Laura Cristoferi, Marco Carbone, Vincenzo Cardinale, Marte L. Høivik, Lise Katrine Engesæter, Sara K. V. Tjønnfjord, Adrian McCann, Per M. Ueland, Mette Vesterhus, Tom H. Karlsen, Henning Grønbæk, Eugenio Gaudio, Konstantinos N. Lazaridis, Annika Bergquist, Espen Melum, Fredrik Bäckhed, Johannes Roksund Hov

**Affiliations:** 1 Norwegian PSC Research Center, Department of Transplantation Medicine, Division of Surgery and Specialized Medicine, Oslo University Hospital, Oslo, Norway; 2 The Wallenberg Laboratory, Department of Molecular and Clinical Medicine, Institute of Medicine, Sahlgrenska Academy, University of Gothenburg, Gothenburg, Sweden; 3 Department of Medicine, Sahlgrenska University Hospital, European Reference Network on Hepatological Diseases (ERN Rare-Liver), Region Västra Götaland, Gothenburg, Sweden; 4 Research Institute of Internal Medicine, Division of Surgery and Specialized Medicine, Oslo University Hospital, Oslo, Norway; 5 Institute of Clinical Medicine, Faculty of Medicine, University of Oslo, Oslo, Norway; 6 Department of Anatomical, Histological, Forensic Medicine and Orthopedics Sciences, Sapienza University of Rome, Rome, Italy; 7 Division of Gastroenterology and Hepatology, Mayo Clinic, Rochester, MN USA; 8 Division of Gastroenterology and Hepatology, School of Medicine, University of Missouri, Columbia, MO USA; 9 Department of Hepatology & Gastroenterology, Aarhus University Hospital, Aarhus, Denmark; 10 Clinical Institute, Aarhus University, Aarhus, Denmark; 11 Hybrid Technology Hub – Centre of Excellence, Institute of Basic Medical Sciences, Faculty of Medicine, University of Oslo, Oslo, Norway; 12 Department of Pathology, Oslo University Hospital, Oslo, Norway; 13 Division of Radiology and Nuclear Medicine, Oslo University Hospital, Oslo, Norway; 14 Section of Gastroenterology, Department of Transplantation Medicine, Division of Surgery and Specialized Medicine, Oslo University Hospital, Oslo, Norway; 15 Department of Medicine Huddinge, Center for Infectious Medicine, Karolinska Institutet, Karolinska University Hospital, Stockholm, Sweden; 16 Department of Medicine, Haraldsplass Deaconess Hospital, Bergen, Norway; 17 Division of Gastroenterology, Center for Autoimmune Liver Diseases, European Reference Network on Hepatological Diseases (ERN RARE-LIVER), IRCCS Fondazione San Gerardo dei Tintori, Monza, Italy; 18 Department of Medicine and Surgery, University of Milano-Bicocca, Milan, Italy; 19 Liver Unit, ASST Grande Ospedale Metropolitano (GOM) Niguarda, Milan, Italy; 20 Department of Translational and Precision Medicine, Sapienza University of Rome, Rome, Italy; 21 Department of Gastroenterology, Division of Medicine, Oslo University Hospital, Oslo, Norway; 22 Bevital AS, Bergen, Norway; 23 Department of Clinical Science, University of Bergen, Bergen, Norway; 24 Unit of Gastroenterology and Nutrition, Department of Medicine Huddinge, Karolinska Institutet, Karolinska University Hospital, Stockholm, Sweden; 25 Department of Clinical Physiology, Sahlgrenska University Hospital, Region Västra Götaland, Gothenburg, Sweden; 26 Novo Nordisk Foundation Microbiome Health Initiative and the National Food Institute, Technical University of Denmark, Kongens Lyngby, Denmark

**Keywords:** Primary sclerosing cholangitis, Chronic inflammation, Metabolism, Biliary tract, Microbiome

## Abstract

Primary sclerosing cholangitis (PSC) is a chronic inflammatory disease of the bile ducts that can lead to biliary cancer and end-stage liver disease. PSC is associated with inflammatory bowel disease and an altered gut microbiota. However, the molecular mechanisms underlying gut–liver interactions in PSC remain poorly characterized. Here we show that the gut microbiota-derived metabolite imidazole propionate (ImP) is a disease driver in PSC. Individuals with PSC have higher circulating ImP levels than individuals with related conditions, and high ImP levels predict reduced survival in PSC. Cholangiocytes exposed to ImP show activated mammalian target of rapamycin complex 1 (mTORC1) signalling and secrete pro-inflammatory and pro-fibrogenic factors. Chronic administration of ImP to mice induces liver inflammation and fibrosis through a p38-dependent mechanism, upstream of mTORC1. We propose that chronic exposure to ImP induces cholangiocyte injury, which alone or in concert with other factors causes clinical worsening of PSC. Therefore, targeting ImP production or signalling may represent therapeutic avenues in PSC.

## Abstract (web summary)

The gut microbial metabolite imidazole propionate (ImP) is shown to promote primary sclerosing cholangitis (PSC) in mice. In humans with PSC, circulating ImP levels are elevated and correlated with poor survival.

## Main

The gut and liver are closely connected: bile from the liver flows through the bile ducts to the intestine and contributes to shaping its environment, while nutrient-rich portal blood from the gut exposes the liver to absorbed dietary, endogenous and microbial compounds. Intestinal diseases are commonly associated with liver co-morbidities, but there is still limited understanding of the molecular interactions of the gut–liver axis. PSC can be considered a model disease for a disturbed gut–liver axis. PSC is an autoimmune disease affecting young adults, characterized by chronic inflammation of the large intrahepatic and extrahepatic bile ducts, leading to progressive fibrotic strictures causing cholestasis and eventually end-stage liver disease^1,2^. Up to 80% of individuals with PSC have concomitant inflammatory bowel disease (IBD), most commonly ulcerative colitis, representing strong evidence of a link between the gut and the liver. Furthermore, PSC is one of the most difficult remaining challenges in hepatology, with no disease-modifying medical therapy available and liver transplantation (LT) as the only treatment option^1^. The median time from diagnosis to LT or death is 13–21 years^1^. A subgroup of people with both PSC and IBD eventually need a colectomy owing to active colitis or colorectal cancer. Interestingly, a proctocolectomy with ileostomy improves the prognosis of the liver disease^3^, which may suggest potential disease-contributing mechanisms from the colonic microbiota.

Chronic inflammatory disorders like IBD or autoimmune liver diseases are associated with altered gut microbiota structure or function^4^. The gut microbiota in PSC is characterized by lower diversity than in healthy individuals^5–7^, and transfer of gut microbiota from individuals with PSC worsens biliary disease in mice^8^. The mechanisms are probably multiple and complex, including specific bacteria or functional alterations in bacterial metabolism^9^. This includes reduced capacity to synthesize vitamin B6 and branched-chain amino acids, with corresponding reduced circulating levels of these essential nutrients, which also associate with a more severe disease course in PSC^10^. Diet and gut microbiota are the strongest determinants of circulating metabolites in humans^11^, and gut microbial diversity predicts levels of circulating metabolites^12^.

In the present study, we aimed to identify microbial metabolites driving PSC by performing comprehensive untargeted metabolomic profiling of peripheral blood, representing biochemical footprints of microbial activity. Metabolomic profiling is challenging in liver diseases because the liver is a key regulator of metabolism, and extensive secondary changes are expected. However, we previously showed that the PSC-associated microbiota alterations persist after LT^7^, and by focusing specifically on metabolites persistently elevated after LT, we found the microbially produced amino acid metabolite ImP to be associated with PSC and its progression. In cholangiocyte organoids derived from people with PSC, we found that ImP promotes a secretory, pro-fibrogenic phenotype. ImP activates the mTORC1 signalling pathway in cholangiocytes and mediates portal inflammation, biliary fibrosis and ductular reaction in the mouse liver in a p38-dependent manner, which is upstream of mTORC1. Taken together, our findings suggest that the dysbiotic gut in PSC favours production of ImP with the potential to impact disease progression.

## Results

### Identification of ImP as a persistent disease modifier in PSC

**Fig. 1:**
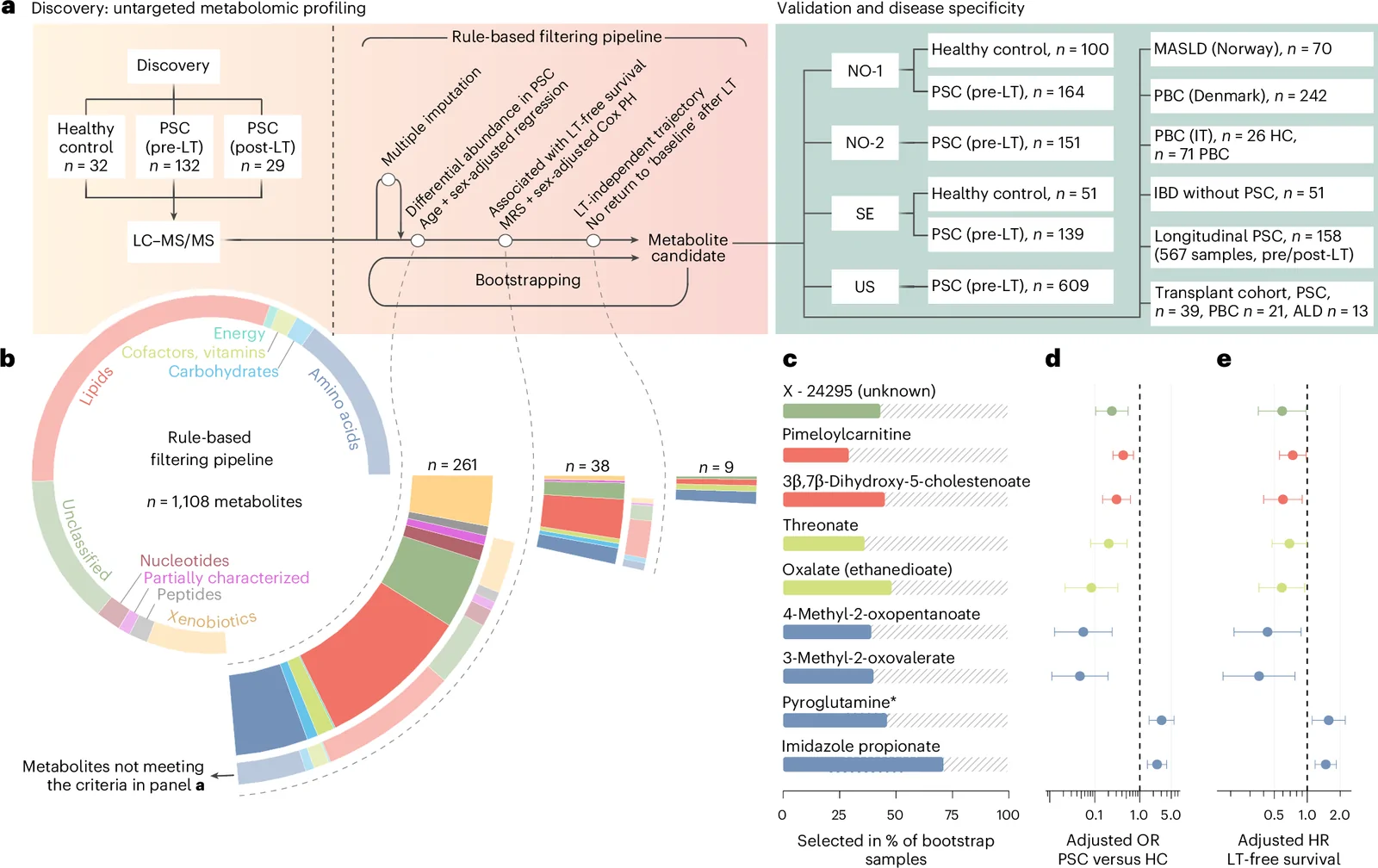
Discovery of ImP as a potential disease modifier in PSC. **a**, Flowchart of the discovery pipeline and cohorts used for targeted validation and evaluation of disease specificity. MRS, Mayo PSC risk score; PH, proportional hazard. **b**, Visualization of the number and classes of metabolites sequentially meeting the criteria at the different steps of the rule-based filtering pipeline shown in **a** (Methods). Faded, thinner bars indicate metabolites excluded at each step. **c**, Frequency with which the candidate metabolites from **b** fulfilled the three criteria in the resampled datasets. **d**, Pooled odds ratios (ORs) and 95% confidence intervals (CIs) from multivariable binary logistic regression analyses on multiply imputed data to predict disease status (PSC versus HCs, HCs as reference) for the selected metabolites. **e**, Pooled hazard ratios (HRs) and 95% CIs from multivariable Cox proportional hazards analyses (adjusted for sex and MRS) on multiply imputed data to predict LT-free survival for the selected metabolites. Source data

**Extended Data Fig. 1:**
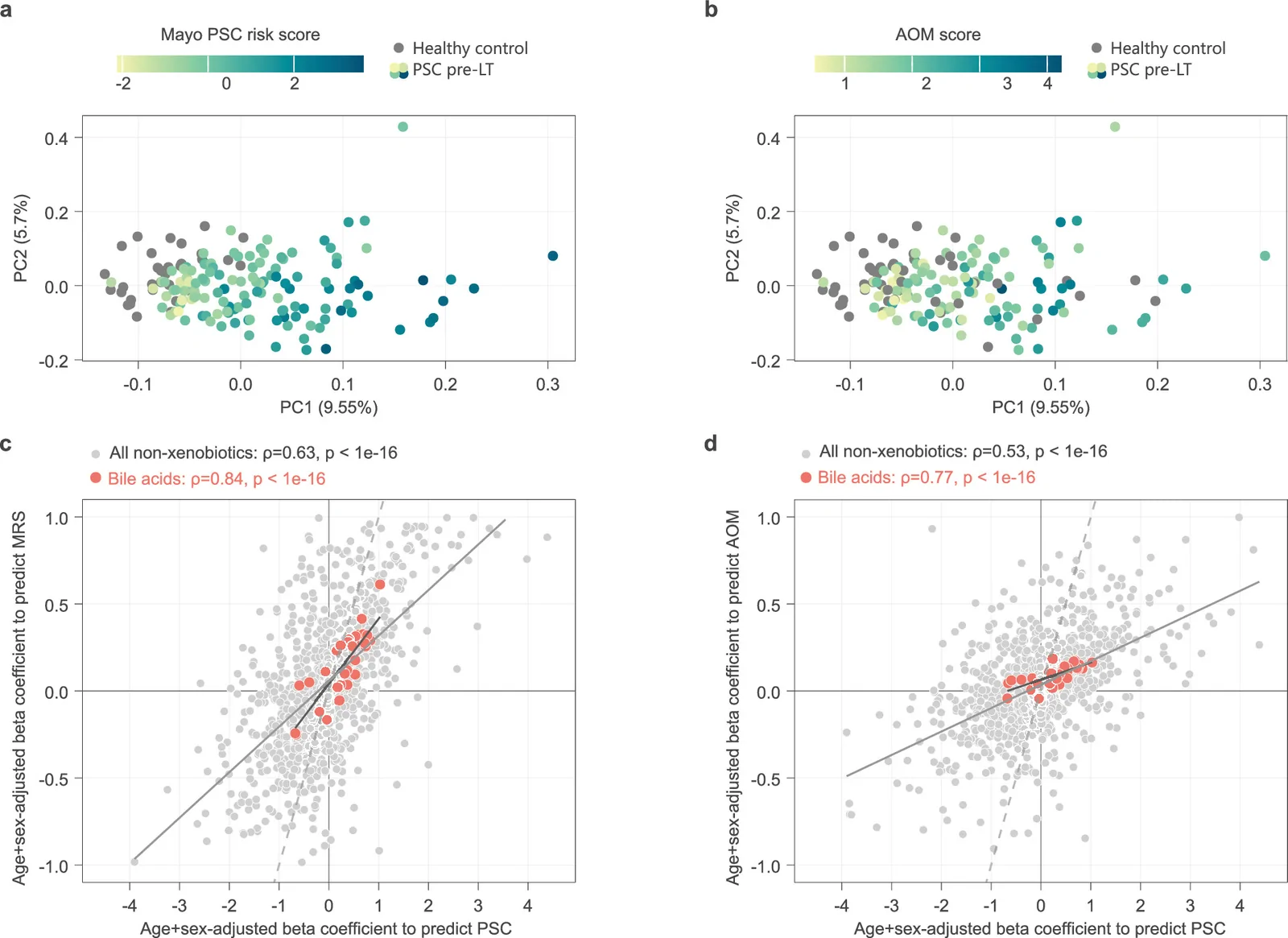
Metabolomic changes in PSC plasma correlate with disease severity. **a-b**. Plots of the first two principal components of metabolomic profiles of samples from healthy controls and PSC pre-liver transplantation (LT) in the untargeted metabolomic profiling (discovery) cohort, derived using principal component analysis. The PSC samples are colored based on their donor’s Mayo PSC risk score (MRS) (**a**) or Amsterdam-Oxford model (AOM) (**b**) scores at time of sampling. **c-d**. Bivariate plots of (i) each metabolite’s age- and sex-adjusted beta coefficient from linear regressions to predict MRS (**c**) or AOM (**d**) and (ii) each metabolite’s age- and sex-adjusted beta coefficient from binary logistic regressions to predict PSC (versus HC). Spearman’s rank correlation (ρ) was calculated for all metabolites and for primary bile acids (PBAs) selectively. HC: healthy controls, LT: liver transplantation, PC: principal component, PSC: primary sclerosing cholangitis, MRS: Mayo risk score, AOM: Amsterdam-Oxford PSC model. Source data

To identify metabolites that can modify PSC, we performed untargeted metabolomic profiling of plasma from individuals with (*n* = 132) and without (*n* = 32) PSC (Fig. 1a and Supplementary Table 1). We found a large shift in the circulating metabolome in individuals with PSC (PSC pre-LT), with 261 out of 1,108 (24%) of the quantified metabolites showing associations with the disease after adjusting for confounding factors and at a false discovery rate (FDR) threshold of 0.01 (Supplementary Table 2). Furthermore, with increasing PSC disease severity as measured by risk scores based on clinical features and liver biochemistry, the metabolomic profiles diverged even further from that of healthy controls (HCs) (Extended Data Fig. 1a,b). Notably, we observed a clear correlation between metabolite associations with PSC and disease score severity (Extended Data Fig. 1c,d), suggesting that a substantial proportion of the variation in the PSC circulating metabolome could be attributed to liver disease itself.

**Extended Data Fig. 2:**
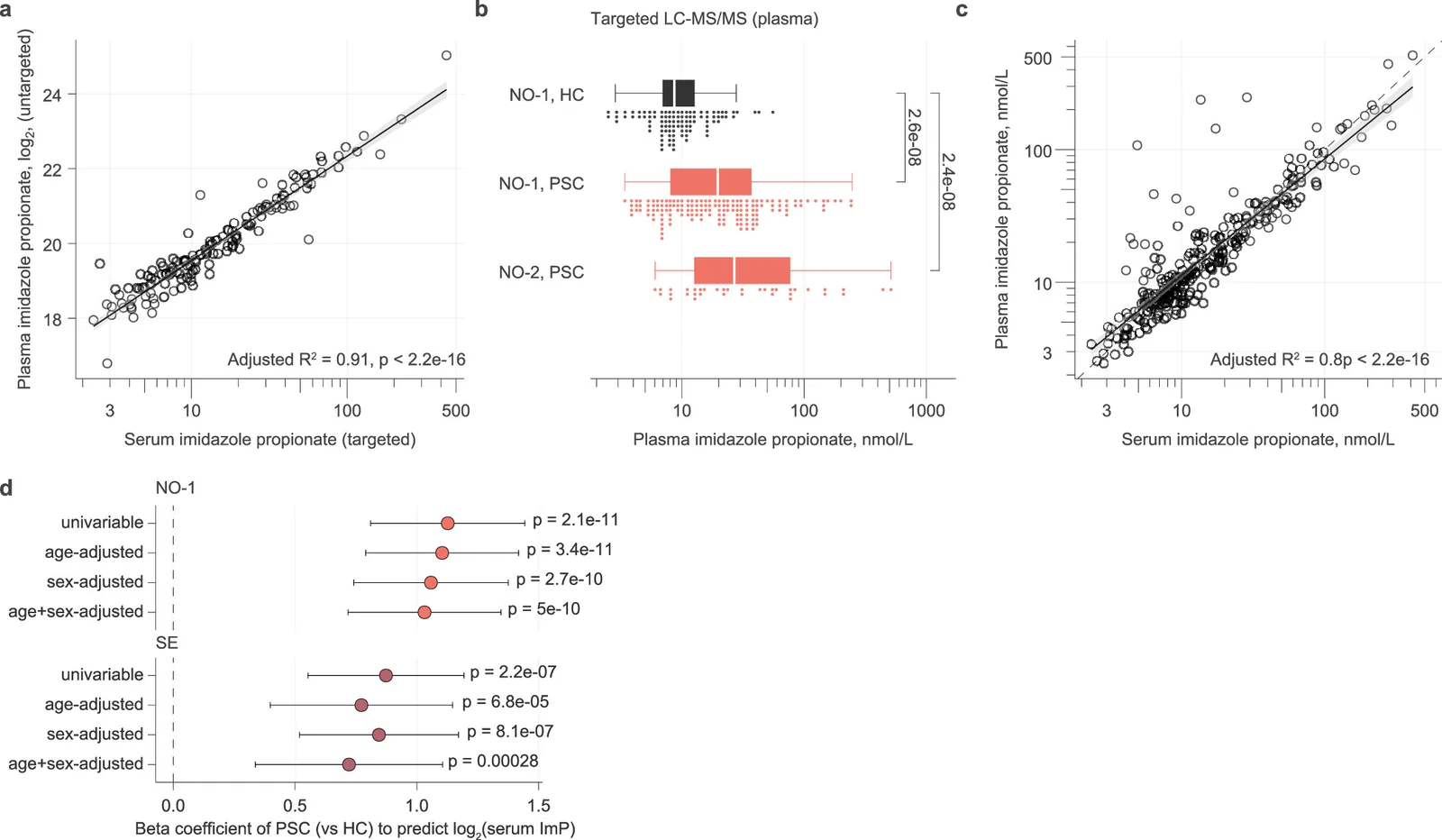
Targeted validation of imidazole propionate, supplementary analyses to Fig. 2. **a**. Shows the relation between scaled, log_2_-transformed imidazole propionate (ImP) levels in the untargeted metabolomic profiling (discovery) cohort and the absolute concentrations of ImP measured in the same blood samples (*n =* 146 plasma pairs) using a targeted method. Linear regression was used to quantify the univariable strength of the association (adjusted R^2^ is shown). The black line indicates the best-fitting linear regression line, and the shaded area indicates the 95% confidence interval around the regression line. **b**. Plasma ImP levels in healthy controls (*n =* 100), NO-1 (*n =* 155) and NO-2 (*n =* 36) cohorts measured using a targeted method (see methods). Points indicate individual ImP measurements. Box plot center lines indicate the median, the bounds of the box indicate the 25th and 75th percentiles, and the whiskers indicate the minimum and maximum values within 1.5 × the interquartile range. Statistical significance was tested using two-sided Mann-Whitney *U-*tests. **c**. Relationship between absolute concentrations of ImP measured in paired serum and plasma from pooled PSC and healthy control samples (*n =* 291 pairs) using the same, targeted method. Linear regression was used to quantify the strength of the univariable association (adjusted R^2^ is shown). The black line indicates the best-fitting linear regression line, and the shaded area indicates the 95% confidence interval around the regression line. **d**. Forest plot showing the univariable and multivariable (adjusted for age and sex) associations between log_2_-transformed ImP and disease status (PSC versus HC, HC as reference). Beta coefficient point estimates are shown as filled circles with error bars corresponding to 95% confidence intervals. Source data

Out of the 261 metabolites associated with PSC, 38 demonstrated a significant association with LT-free survival after adjusting for sex and disease severity (Fig. 1b and Supplementary Table 3). With the rationale that metabolites contributing to PSC should be produced independently of liver function, we subsequently retained only metabolites with similar concentrations before and 5 years after LT (Supplementary Table 4). This filtering criterion yielded nine candidate metabolites (Supplementary Table 5). To test the internal validity of these metabolites, we bootstrapped the complete filtering pipeline. ImP, a microbially derived amino acid derivative previously found to associate with type 2 diabetes^13^, heart failure^14^ and wound healing^15^, was the most frequently selected metabolite (71%) in the bootstrap samples and was chosen for further validation (Fig. 1c). To confirm elevated levels of ImP in PSC, we used targeted liquid chromatography with tandem mass spectrometry (LC–MS/MS) analysis in serum samples from an expanded Norwegian cohort, denoted NO-1, consisting of 164 PSC pre-LT and 100 HC cross-sectional samples, 117 and 29 of which had also been part of the untargeted metabolomics experiment. Additionally, we examined serum from an independent Norwegian PSC pre-LT cohort, designated NO-2 (*n* = 151; Supplementary Tables 6 and 7). The relative ImP levels were strongly correlated with targeted measurements (adjusted *R*^2^ = 0.91; Extended Data Fig. 2a).

**Fig. 2:**
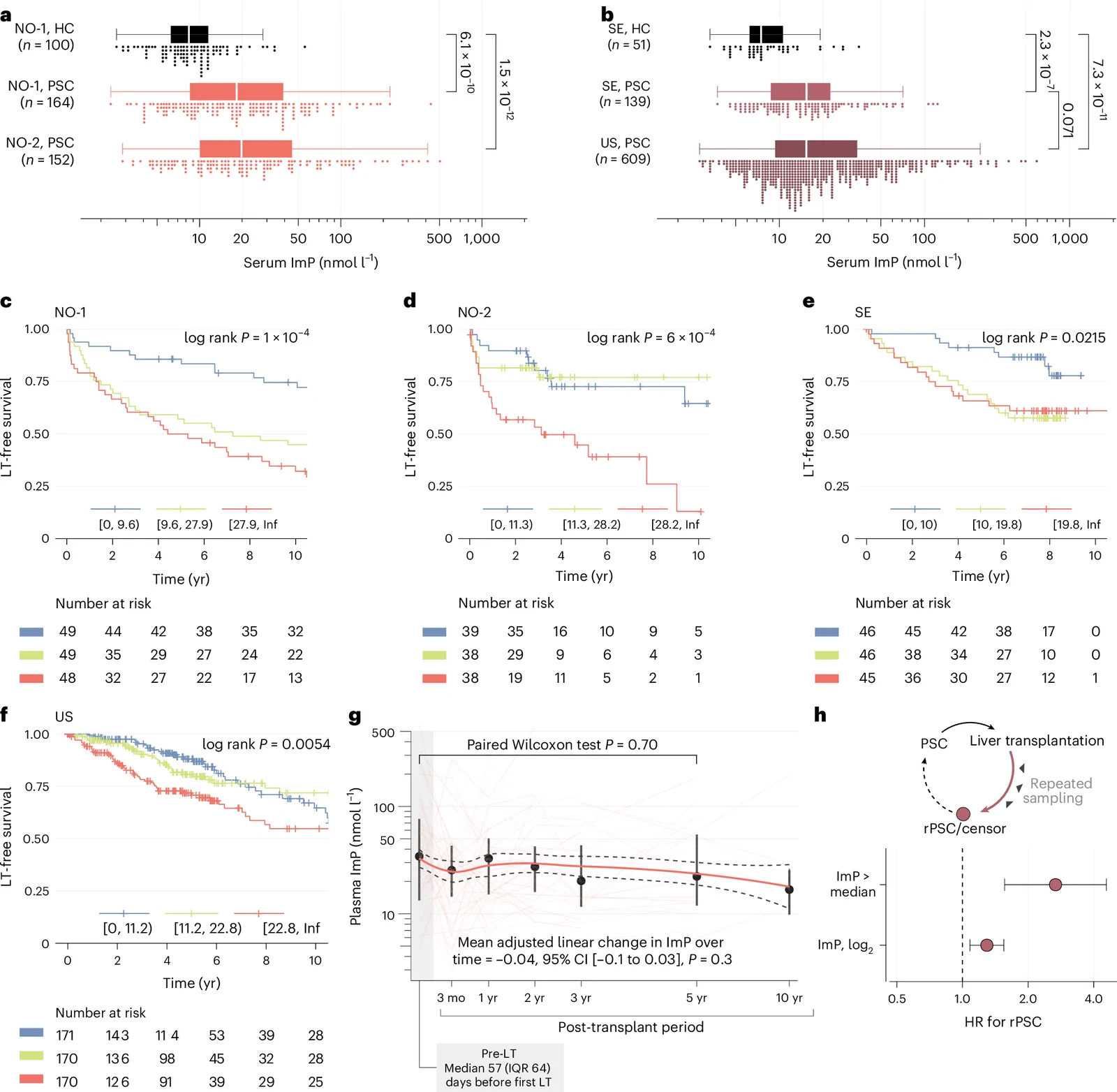
Targeted validation of the rule-based filtering pipeline and ImP as a disease modifier in PSC. **a**, ImP levels in serum samples from HCs and people with PSC in the expanded Norwegian cohort NO-1 and independent targeted cohort NO-2, measured using targeted LC–MS/MS. **b**, Serum ImP levels in HCs and people with PSC in external validation cohorts from Sweden (SE; measured using a different targeted LC–MS/MS protocol) and the USA (US). Differences between groups were tested using two-sided Mann–Whitney *U*-tests. In **a** and **b**, points represent individual ImP measurements. Box plot centre lines indicate the median, the bounds of the box indicate the 25th and 75th percentiles and the whiskers indicate the minimum and maximum values within 1.5× the interquartile range (IQR). **c**–**f**, LT-free survival curves of individuals with PSC in NO-1, NO-2, SE and US cohorts, stratified by ImP tertiles. Differences in overall survival distributions were tested using log-rank tests. **g**, Individual longitudinal trajectories of ImP in the longitudinal PSC cohort, including only samples taken before an rPSC diagnosis (*n* = 403 samples from 143 individuals). A LOESS curve (red line with dotted lines indicating 95% CIs) was fit to the data, and median and IQRs are shown for each timepoint in black. The average adjusted change in log(ImP) over time was estimated using a mixed-effects model, with patient ID as a random effect and age and sex as fixed covariates (model estimates are shown in the plot). The model allowed for correlation in both the intercept and the effect of time for each subject by specifying a first-order autoregressive structure. A paired Wilcoxon signed-rank test was performed to test for differences in ImP levels in paired samples from pre-LT and 5-year samples. **h**, Schematic illustration of rPSC as a time-to-event outcome in post-transplant PSC; triangles indicate serial sampling. The forest plot shows univariable HR point estimates and 95% CIs for ImP (dichotomized by the median and log_2_-transformed) modelled as time-varying predictors of rPSC (*n* = 300 samples from 119 individuals; 75 rPSC diagnoses). Source data

We confirmed elevated ImP levels in PSC (Fig. 2a and Extended Data Fig. 2b), and we replicated this observation in external validation cohorts from Sweden (SE; *n* = 139 PSC and *n* = 51 HCs) and the USA (US; *n* = 609 PSC; Fig. 2b). In individuals with paired serum-plasma samples, ImP levels were strongly correlated (291 pairs, adjusted *R*^2^ = 0.80; Extended Data Fig. 2c), showing that both matrices can be used. In cross-sectional cohorts with available control populations, ImP demonstrated an association with PSC after accounting for age and sex (Extended Data Fig. 2d).

Elevated levels of ImP were consistently linked to a decrease in LT-free survival across all four cross-sectional PSC cohorts (Fig. 2c–f; baseline characteristics in Supplementary Table 8). Furthermore, in a longitudinal cohort in which individuals with PSC were sampled before and serially after LT (*n* = 567 samples from 158 individuals)^16^, plasma ImP levels were highest immediately before LT (Fig. 2d), falling slightly after LT to the levels seen in the cross-sectional NO-1 and NO-2 PSC cohorts. These analyses suggest that ImP levels are largely unaffected by LT, thus confirming our discovery in the untargeted metabolomics (discovery) cohort. Notably, recurrent PSC (rPSC) after LT is a significant clinical problem^1^. Higher circulating ImP levels were associated with an increased risk of rPSC after LT (hazard ratio per doubling = 1.18, 95% CI = 1.01–1.39, *P* = 0.041; hazard ratio for ImP > median = 1.98, 95% CI = 1.24–3.17, *P* = 0.0041; Fig. 2h) (Supplementary Table 9).

### ImP levels are elevated in PSC compared to other liver and inflammatory conditions

**Fig. 3:**
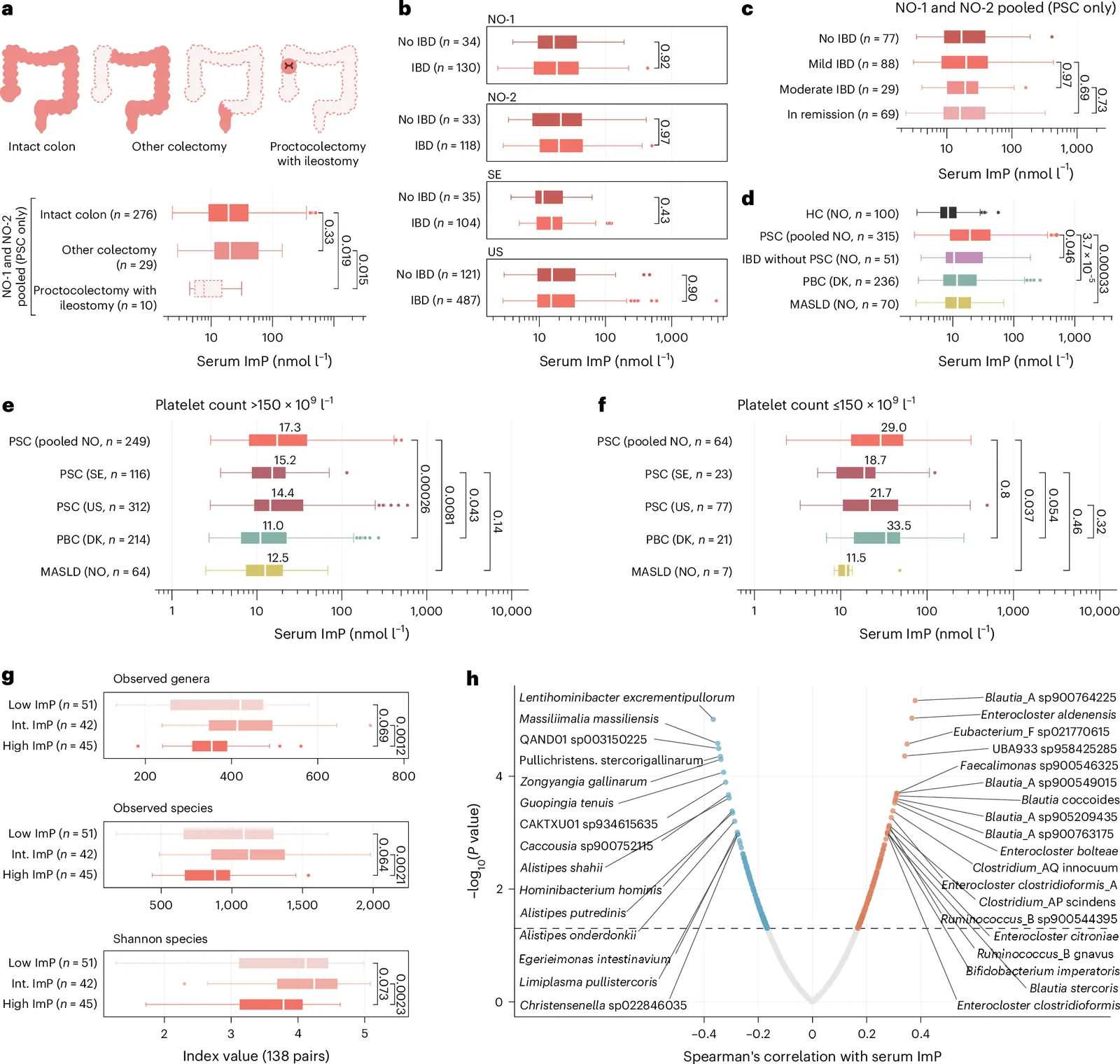
Disease specificity of ImP. **a**, Schematic illustration of different types of colectomy, and distributions of serum ImP in people with PSC by colectomy status at the time of sampling (NO-1 and NO-2 cohorts pooled). ‘Other colectomy’ encompasses other types of colectomies, including colectomy with ileorectal anastomosis or ileal pouch–anal anastomosis. **b**, Serum ImP in the PSC pre-LT cohorts by IBD concurrency. **c**, Serum ImP in the NO-1 and NO-2 cohorts pooled, by IBD activity at time of sampling. **d**, Serum ImP in individuals with IBD without PSC, PBC and metabolic dysfunction-associated steatotic liver disease (MASLD), alongside HCs and individuals with PSC from the NO-1 and NO-2 cohorts (same data used to produce Fig. 2a). **e**,**f**, Serum ImP in various liver diseases stratified by platelet counts (above versus below 150 × 10^9^ l^−1^). The numbers above the box plots indicate medians. **g**, Observed gut microbial genera, species and Shannon species in stool samples from people with PSC who had a blood sample taken within ±14 days of stool collection, stratified by ImP categories (same thresholds as in Fig. 2f). **h**, Correlations between individual gut bacterial species and serum ImP levels were calculated using Spearman’s rank correlation, with the *x* axis showing Spearman’s rank correlation and the *y* axis showing the corresponding unadjusted *P* value. Associations with raw *P* values of <0.05 are coloured blue or red, while species with text annotation indicate a Benjamini–Hochberg-adjusted *P* < 0.05. For **a**–**g**, differences in distributions were tested using two-sided Mann–Whitney *U*-tests. In **a**–**g**, box plot centre lines indicate the median, the bounds of the box indicate the 25th and 75th percentiles and the whiskers indicate the minimum and maximum values within 1.5× the IQR. Data points beyond the whiskers are plotted individually as outliers. Source data

ImP is known to be produced by commensal microbiota, but the key factors influencing its aberrant levels in PSC remain unknown^13,17^. To address this knowledge gap, we first examined serum ImP levels in individuals with PSC stratified by prior colectomy type. Individuals who had undergone a proctocolectomy with ileostomy had reduced, but not abolished, serum ImP levels compared to those with other types of colectomies and reconstructions, including ileal pouch–anal anastomoses and those who had not undergone colectomy (Fig. 3a), suggesting a contribution of colon-resident microbiota to circulating ImP.

ImP levels were consistent among people with PSC regardless of the presence of concurrent IBD and the IBD disease activity (Fig. 3b,c). However, in individuals with longstanding IBD but without PSC (Supplementary Table 10), the median concentration of ImP in serum was 10.5 nM. This was higher than in HCs (8.5 nM) but substantially lower than in the Norwegian PSC pre-LT cohorts (19.1 nM in the pooled Norwegian cohort; Fig. 3d).

**Extended Data Fig. 3:**
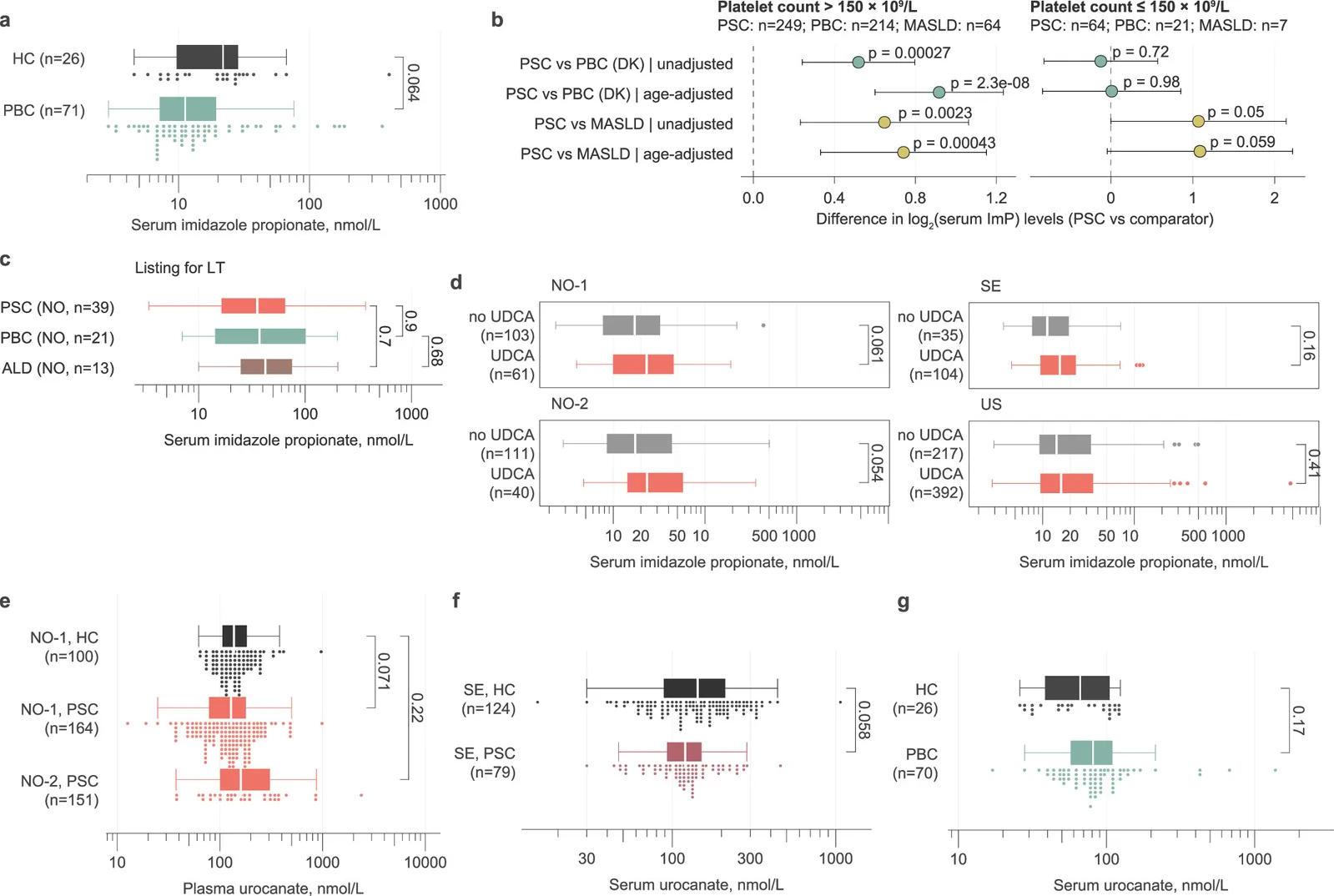
Disease specificity of imidazole propionate, supplementary analyses to Fig. 3. **a**. Serum levels of imidazole propionate (ImP) in people with primary biliary cholangitis (PBC) and healthy controls from the Italian PBC cohort. **b**. Univariable and age-adjusted linear regression estimates (points indicate beta coefficient point estimates and error bars indicate 95% confidence intervals) of the association between disease group (PSC versus PBC or MASLD) and log_2_(ImP), stratified by platelet count (above versus below 150×10^9^/L). Regression coefficients for PSC indicate the adjusted difference in log_2_(ImP) compared with PBC or MASLD. **c**. Distributions of serum ImP in people with PSC, PBC and alcohol-related liver disease (ALD) listed for liver transplantation (LT). **d**. Distributions of ImP in people with PSC in the PSC pre-LT cohorts by ursodeoxycholic acid (UDCA) treatment. **e**. Distributions of plasma urocanate, measured using a targeted method, in serum samples from the Norwegian PSC pre-LT cohorts. **f**. Serum levels of urocanate in people with PSC and healthy controls in the Swedish PSC pre-LT cohort. **g**. Serum levels of urocanate in people with primary biliary cholangitis (PBC) and healthy controls from the Italian PBC cohort. For panels **a**, **c**, **d**, **e**, **f** and **g**, box plot center lines indicate the median, the bounds of the box indicate the 25th and 75th percentiles, and the whiskers indicate the minimum and maximum values within 1.5 × the interquartile range. For panels **c** and **d**, data points beyond the whiskers are plotted individually as outliers. Aside from in panel **b**, statistical significance was tested using two-sided Mann-Whitney *U*-tests. Source data

In other liver diseases, serum ImP was also lower than in PSC (Fig. 3d and Extended Data Fig. 3a). This included primary biliary cholangitis (PBC), an autoimmune liver disease that affects the small bile ducts of the liver (median ImP = 11.9 nM and 11.0 nM in a Danish and an Italian cohort, respectively; Supplementary Tables 11 and 12) and metabolic dysfunction-associated liver disease (MASLD; 12.0 nM; Supplementary Table 13). Positive correlations between increasing disease stage and circulating ImP levels were found both in PSC (Spearman’s ρ with the clinical Mayo PSC risk score = 0.45, *P* = 2.3 × 10^−17^; ρ = 0.36, *P* = 1.4 × 10^−5^; and ρ = 0.27, *P* = 3.0 × 10^−8^, in the NO, SE and US cohorts, respectively) (Fig. 3f), and PBC, with a positive correlation with liver stiffness measured by elastography (Spearman’s ρ with elastography = 0.24, *P* = 0.0002). Given that these cross-disease comparisons could be confounded by differences in disease severity and the measures of disease severity, we stratified by platelet counts, which were available for the majority of participants. A platelet count below 150 × 10^9^ l^−1^, commonly seen in advanced liver diseases with portal hypertension, was used as a surrogate marker and was associated with generally higher serum ImP concentrations (Fig. 3e). Among individuals with normal platelet counts, ImP levels were significantly higher in PSC than in PBC and MASLD, but not in those with low platelet counts (Fig. 3e), a finding supported by age-adjusted linear models (Extended Data Fig. 3b). The observation of higher ImP levels in advanced liver disease was supported by samples measured at time of listing for LT, in which ImP levels were similarly elevated in a cohort of transplant candidates with PSC, PBC or alcohol-related liver disease (Extended Data Fig. 3c). Overall, this suggests that ImP was clearly higher in PSC than other diseases in earlier stages of disease, and there was a convergence towards high ImP concentrations in very advanced liver disease, irrespective of aetiology.

Liver conditions such as PBC, MASLD and alcohol-related liver disease are, like PSC, known to involve gut microbial alterations^4,18^, and cirrhosis itself—in particular in a decompensated state—is associated with shared gut environmental and microbial alterations across the spectrum of liver diseases^19^. The particularly elevated ImP in PSC may therefore reflect PSC-specific microbial changes that are later masked by cirrhosis and portal hypertension-driven microbial alterations. To investigate the microbial contribution to ImP, we analysed temporally matched blood and stool samples (taken up to 14 days apart) from 138 individuals with PSC. Here, high ImP was associated with reduced gut microbial diversity, including a lower number of observed genera and species, and reduced Shannon diversity (Fig. 3e). At an FDR threshold of 0.05, we identified 19 species that were positively correlated with circulating ImP levels (Supplementary Table 14), including *Enterocloster clostridioformis* and *Enterocloster boltae*, both previously shown to be increased in PSC^10^, and *Ruminococcus gnavus*, previously linked to ImP production^17^.

There were no substantial differences in ImP levels between users and non-users of ursodeoxycholic acid, a commonly prescribed drug in PSC (Extended Data Fig. 3d). Levels of urocanate, which can be produced both by the host and bacteria and is reduced exclusively by bacterial urocanate reductase (urdA) into ImP^13^, were comparable between individuals with PSC and HCs (Extended Data Fig. 3e,f), and individuals with PBC and HCs (Extended Data Fig. 3g), supporting that microbial activity rather than substrate availability affect the circulating levels of ImP in humans.

### ImP activates human cholangiocytes and induces a pro-inflammatory and pro-fibrotic phenotype

**Fig. 4:**
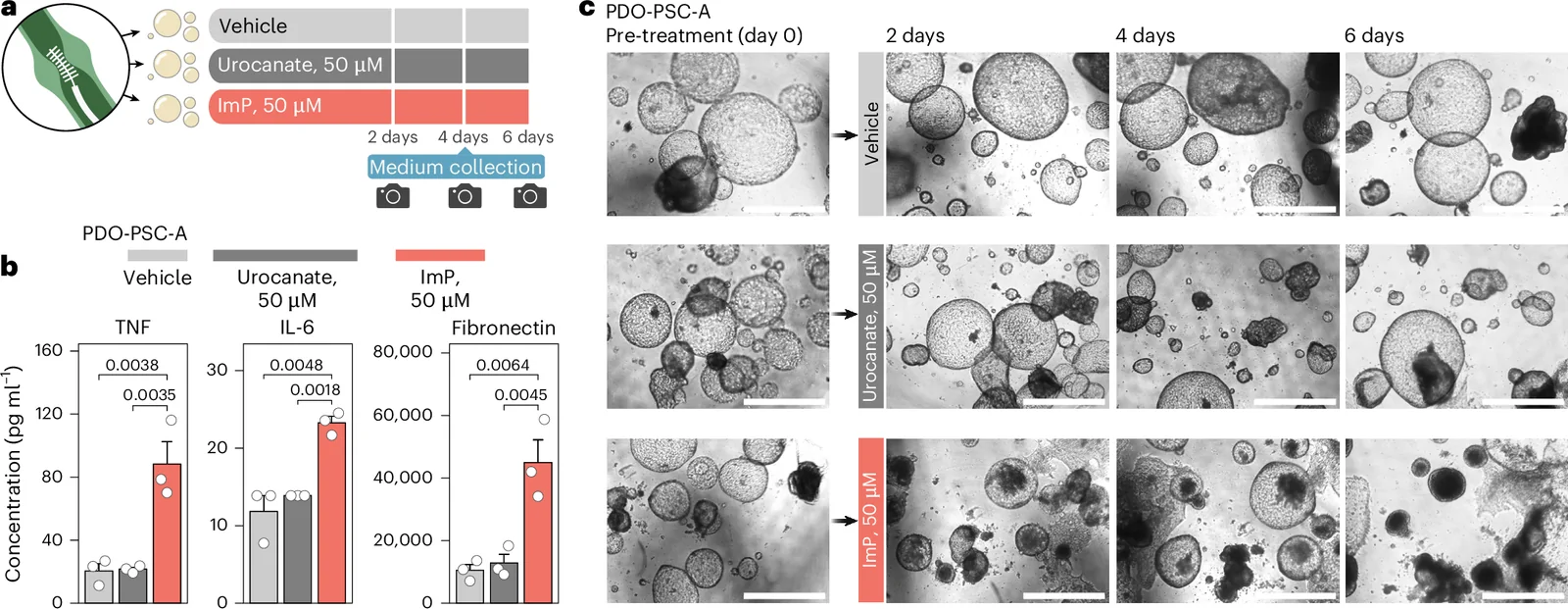
Chronic administration of ImP induces duct inflammation and fibrosis in human-derived cholangiocyte organoids. **a**, Schematic representation of the experimental set-up. **b**. Concentrations of tumour necrosis factor (TNF), interleukin-6 (IL-6) and fibronectin in conditioned medium collected at day 4 from cholangiocyte organoids derived from a donor with PSC (PDO-PSC-A), after the organoids were treated for 4 days with ImP (50 µM), urocanate (Uro, 50 µM) or vehicle. **c**, Light microscopy time-series images of PDO-PSC-A organoids at baseline (day 0, pre-treatment) and after 2, 4 and 6 days of treatment with ImP (50 µM), Uro (50 µM) or vehicle. The organoid experiment was performed in technical triplicate; bars represent mean values; error bars, s.e.m. Statistical significance was calculated using ANOVA with Tukey’s honestly significant difference post hoc test. Data on additional markers and experimental models from two additional donors (yielding similar results) are shown in Supplementary Fig. 1. Scale bars, 100 µm. PDO, patient-derived organoid. Source data

Activated cholangiocytes secrete pro-inflammatory and pro-fibrotic substances, a feature believed to occur early in the cascading development of cholangiopathies like PSC^20^. To test whether ImP could function as a cholangiocyte-activating substance, we cultured endoscopic retrograde cholangiopancreatography-derived cholangiocytes from three people with PSC as organoids ex vivo and treated them with ImP or urocanate for 6 days (Fig. 4a). The concentrations of pro-inflammatory and pro-fibrotic cytokines were elevated in conditioned media from ImP-treated organoids compared to urocanate and vehicle (Fig. 4b and Supplementary Fig. 1). In addition, ImP treatment impaired the ability of the organoids to maintain a spherical morphology (Fig. 4c), suggesting that ImP directly interacts with cholangiocytes.

### Chronic administration of ImP induces biliary inflammation and liver fibrosis in mice

**Fig. 5:**
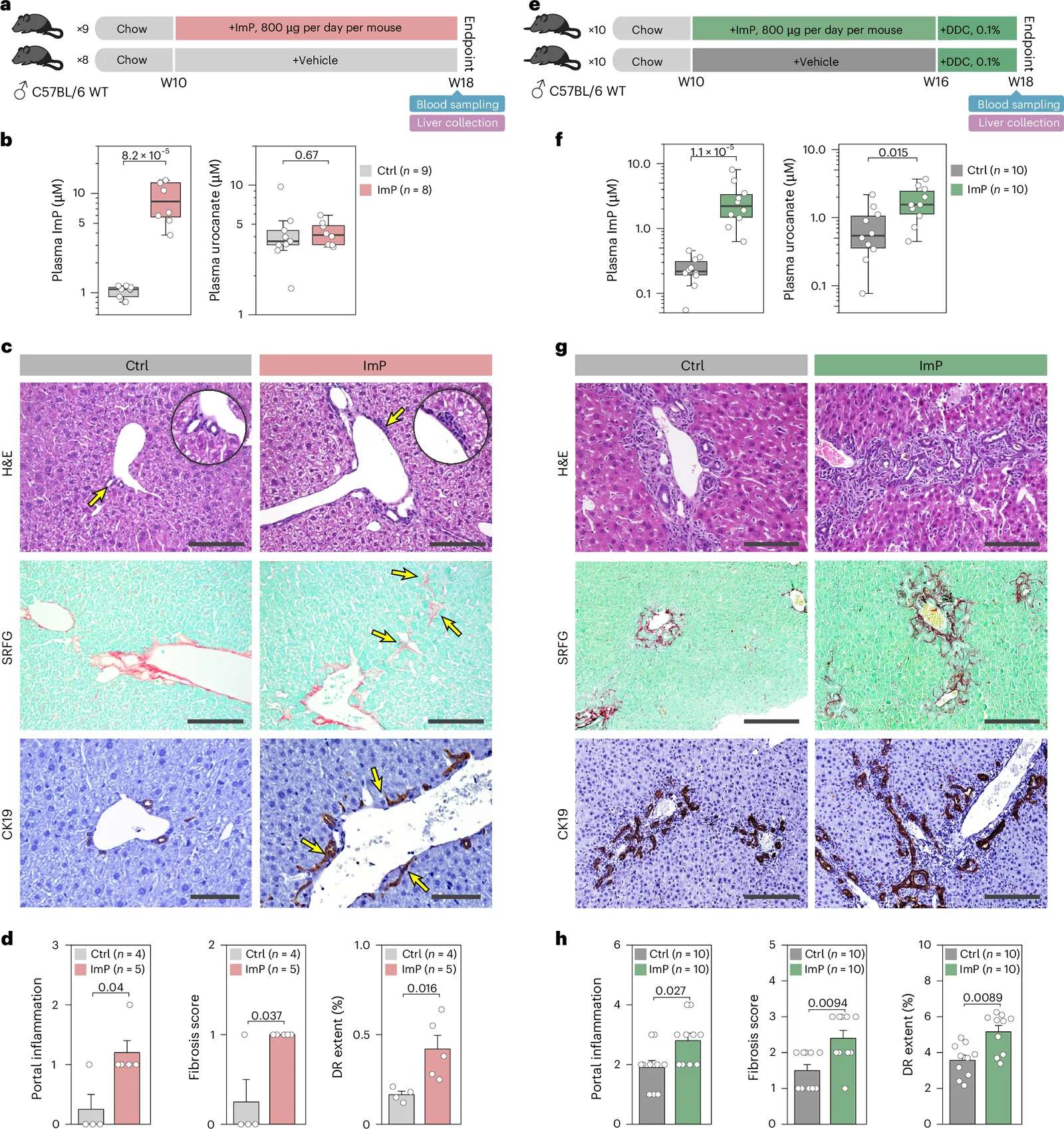
Chronic administration of ImP induces duct inflammation and fibrosis in vivo. **a**, Schematic representation of the experimental design using C57BL/6J wild-type (WT) mice on a chow diet (*n* = 8 or 9 mice per group). **b**, Plasma levels of ImP and urocanate in WT mice treated with vehicle (Ctrl) or ImP for 8 weeks on a chow diet. **c**,**d**. Representative images (**c**) and relative quantification (**d**) for haematoxylin and eosin (H&E), Sirius red fast green (SRFG) and immunohistochemistry for CK19 in livers of mice treated without or with ImP on a chow diet. Scale bars, 100 μm (H&E, SRFG) and 50 μm (CK19). Arrows indicate areas of portal inflammation (magnified in the circles; H&E), collagen fibres (SRFG) or ductular reaction (DR; CK19). Among the eight and nine mice per group, four and five were randomly selected for liver histology analyses. **e**, Schematic representation of the experimental design using WT mice on a 0.1% DDC diet (*n* = 10 per group). **f**, Plasma levels of ImP and urocanate in WT mice treated with vehicle (Ctrl) or ImP for 8 weeks on a 6-week chow diet followed by a 2-week DDC diet. **g**,**h**, Representative images and relative quantification for H&E, SRFG and immunohistochemistry for CK19 in livers of mice treated with or without ImP on a chow diet. Scale bars, 100 μm (H&E, SRFG) and 50 μm (CK19). For **b** and **f**, box plot centre lines indicate the median, the bounds of the box indicate the 25th and 75th percentiles and the whiskers indicate the minimum and maximum values within 1.5× the IQR. For **d** and **h**, bars represent the mean values; error bars, s.e.m. *P* values were calculated using two-sided Mann–Whitney *U-*tests. Source data

**Extended Data Fig. 4:**
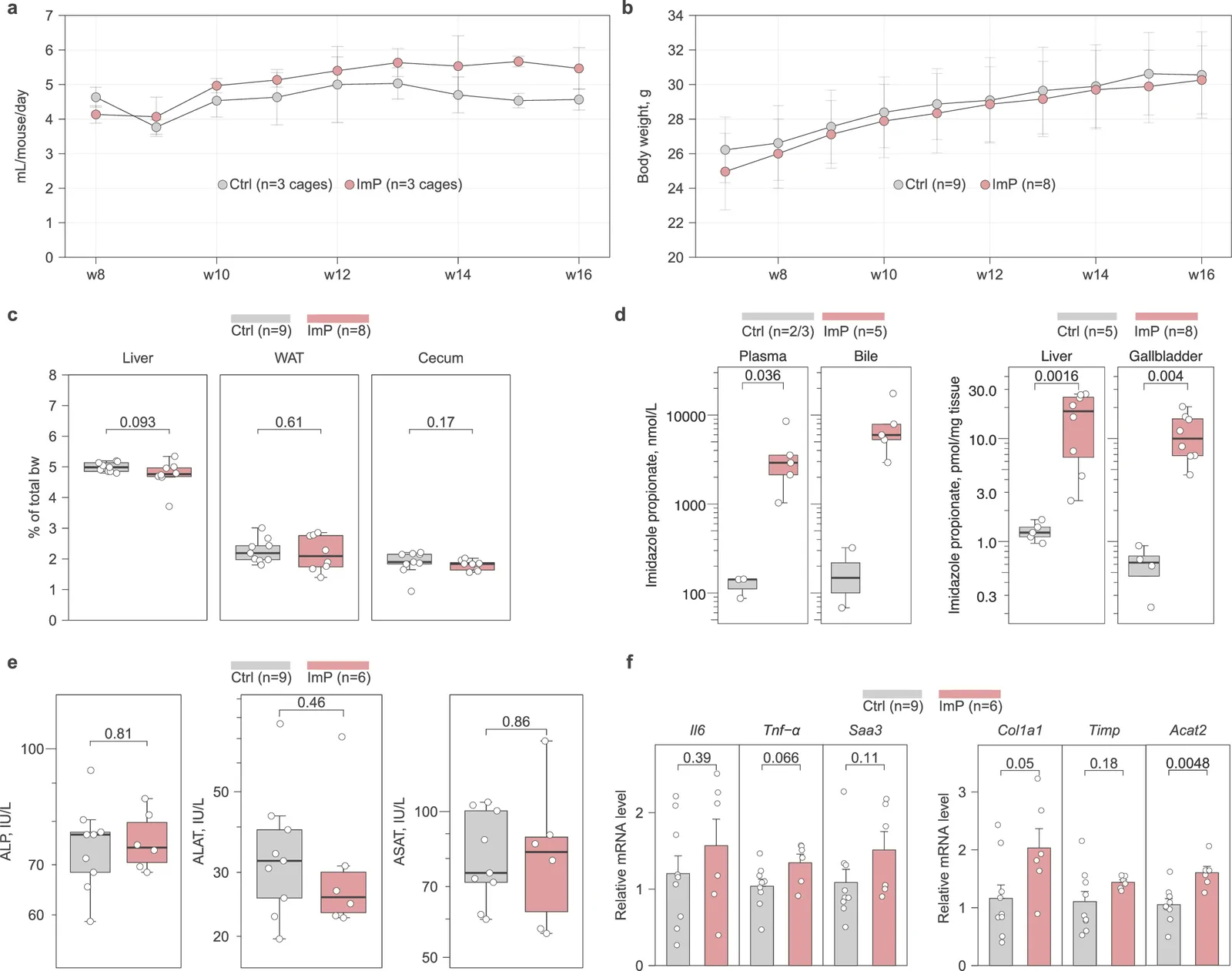
Anthropometric parameters, serum liver enzyme and gene expression in the livers of wild type mice treated with and without imidazole propionate on a chow diet. **a-b**. Water intake and body weight in 8-week-old mice treated with vehicle (Ctrl) or imidazole propionate (ImP) on a chow diet (*n =* 8 and 9 per group with data shown per cage). **c**. Organ weights (as percentage of body weight) at sacrifice in mice treated with or with ImP. **d**. Levels of ImP in plasma, bile, gallbladder and liver in mice treated with ImP for 8 weeks (independent cohort from the other panels in this figure, n = 3 and 8 per group). **e**. Serum levels of ALP, ASAT and ALAT at sacrifice. For panels **c**-**e**, box plot center lines indicate the median, the bounds of the box indicate the 25th and 75th percentiles, and the whiskers indicate the minimum and maximum values within 1.5 × the interquartile range. **f**. Relative mRNA levels of inflammation- and fibrosis-related genes in mouse liver extracts. Bars represent the mean, and error bars represent the standard error of the mean (SEM). For panels **c**-**f**, p-values were calculated using two-sided Mann-Whitney *U*-tests. ALP: alkaline phosphatase, ASAT: aspartate aminotransferase, ALAT: Alanine aminotransferase, Il6: interleukin 6, Saa3: serum amyloid 3, Tnfα: tumor necrosis factor alpha, Acat2: Acetyl-CoA acetyltransferase, Col1a1: alpha-1 type I collagen, Timp: TIMP metallopeptidase inhibitor 1, WAT: white adipose tissue. Source data

**Extended Data Fig. 5:**
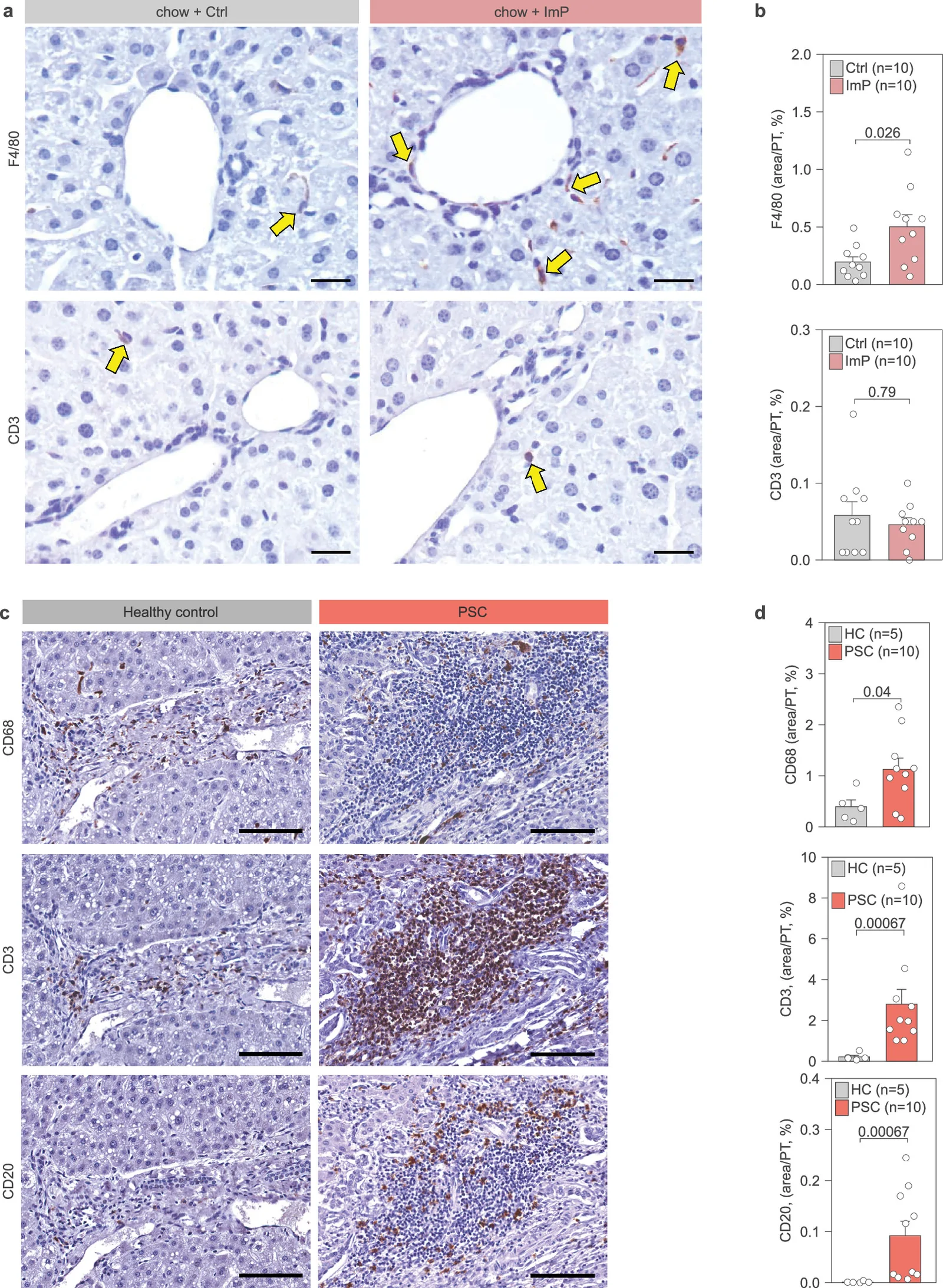
Immune cell staining in mice treated with imidazole propionate and in individuals with and without PSC. **a-b**. Representative pictures and relative quantification of F4/80 and CD3 in wild type mice treated with vehicle (Ctrl) or imidazole propionate (ImP, *n* = 10 per group; same mice used for Fig. 7). Scale bars = 20 μm. **c-d**. Representative pictures and relative quantification of CD68, CD3 and CD20 in human healthy controls and individuals with PSC (*n =* 6 and 10 per group, same individuals as in Fig. 6**panels g-h**). Scale bars = 100 μm. Bars represent the mean, and error bars represent the standard error of the mean (SEM). P-values were calculated using two-sided Mann-Whitney *U*-tests. Source data

Next, we investigated whether chronic ImP exposure induces cholangiocyte injury in vivo by administering ImP to 6-week-old wild-type C57BL/6J mice in the drinking water for 8 weeks (Fig. 5a). There were no significant differences in water intake, body weight or organ weights of the mice by treatment group (Extended Data Fig. 4a–c). Plasma concentrations of ImP, but not urocanate, increased in ImP-treated mice compared to vehicle-treated controls (Ctrl) (Fig. 5b). ImP-treated mice also demonstrated elevated ImP levels in liver tissue, gallbladder tissue and bile, as measured in a limited number of mice (Extended Data Fig. 4d), indicating biliary excretion and ImP exposure of cholangiocytes. Quantifications of villi and crypt length and Ki67 staining (a marker of epithelial cell proliferation) indicated no differences in small and large intestine morphology (Supplementary Fig. 2). By contrast, ImP-treated compared with Ctrl mice exhibited increased liver portal inflammation (Fig. 5c). This finding was supported by increased F4/80-positive macrophages in the livers of ImP-treated mice, consistent with macrophage accumulation in portal areas that was also evident in human PSC liver sections (Extended Data Fig. 5a–d). No differences were observed in CD3^+^ T cell infiltration between the treatment groups (Extended Data Fig. 5a,b), which contrasts with human PSC, where there was evidence of increased infiltration of T cells (CD3) and B cells (CD20) (Extended Data Fig. 5c,d). ImP-treated mice also showed increased histological features of liver fibrosis, especially in the portal areas (Fig. 5c,d), a finding we confirmed using an artificial intelligence-based, quantitative analysis of the same liver sections (Supplementary Fig. 3). Moreover, the proportion of proliferating cytokeratin 19-positive (CK19^+^) cholangiocytes was increased in ImP-treated compared with Ctrl mice (Fig. 5c). Despite these histological changes, serum alkaline phosphatase (ALP) as well as aspartate aminotransferase (ASAT) and alanine aminotransferase (ALAT) levels were comparable between groups. The expression levels of pro-inflammatory and pro-fibrotic genes tended to be elevated in whole livers from ImP-treated mice (Extended Data Fig. 4d–f).

**Extended Data Fig. 6:**
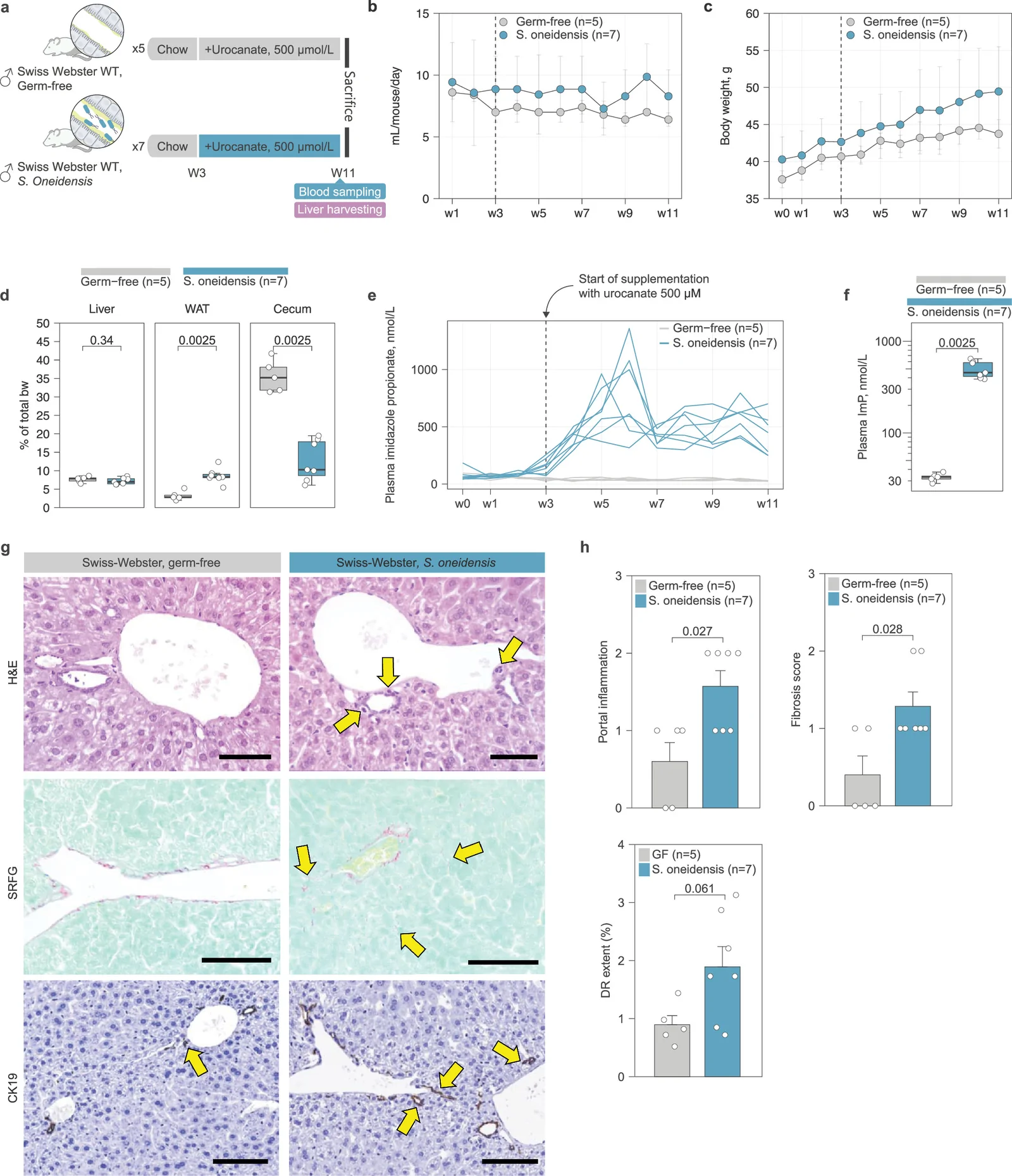
Imidazole propionate produced by *Shewanella oneidensis* impacts bile ducts inflammation and fibrosis in colonized mice. **a**. Schematic representation of the colonization experiment with *Shewanella oneidensis*. **b-c**. Water intake and body weight in 11-week-old gnotobiotic mice colonized with *S. oneidensis* and germ-free mice (*n* = 4 and 7 per group). **d**. Organ weights (as percentage of body weight) at sacrifice in *S. oneidensis-*colonized and germ-free mice. **e-f**. Imidazole propionate (ImP) levels during treatment and at sacrifice in *S. oneidensis-*colonized and germ-free mice. The dotted line in (**e**) refers to the start of supplementation with urocanate (500 µM) in the drinking water. Values in (**f**) are median concentrations per mouse for weeks 4-11. **g-h**. Representative images and relative quantification for hematoxylin and eosin (H&E), Sirius red fast green (SRFG) and immunohistochemistry for cytokeratin 19 (CK19) in livers of *S. oneidensis-*colonized and germ-free mice. Scale bars = 100 μm (H&E, SRFG) and 50 μm (CK19). Arrows indicate areas of portal inflammation (H&E), collagen fibers (SRFG) or ductular reaction (CK19). Bars represent the mean, and error bars represent the standard error of the mean (SEM). For panels d and f, box plot center lines indicate the median, the bounds of the box indicate the 25th and 75th percentiles, and the whiskers indicate the minimum and maximum values within 1.5 × the interquartile range. P-values were calculated using two-sided Mann-Whitney *U-*tests. Source data

To test whether physiologically relevant, gut-microbiota-derived ImP could induce cholangiocyte injury in vivo, we colonized germ-free Swiss Webster mice, an outbred strain well suited for bacterial colonization^21^, with *Shewanella oneidensis* and administered urocanate to the mice through their drinking water (Extended Data Fig. 6a). *S.* *oneidensis* is known to express the rate-limiting bacterial UrdA^22^, which is required for ImP synthesis from urocanate. No differences were observed in body weight or organ weights between germ-free and *S.* *oneidensis*-colonized mice, except for a significant reduction in caecum size, a well-documented consequence of microbial colonization (Extended Data Fig. 6b–d). Colonization with *S.* *oneidensis* increased ImP plasma concentrations into the micromolar range (Extended Data Fig. 6e,f). Histological analysis revealed that *S.* *oneidensis*-colonized mice displayed statistically significant increases in portal inflammation and liver fibrosis as well as increased ductular reaction compared with germ-free controls, recapitulating the major pathological features observed following chronic ImP administration in drinking water (Extended Data Fig. 6g,h).

**Extended Data Fig. 7:**
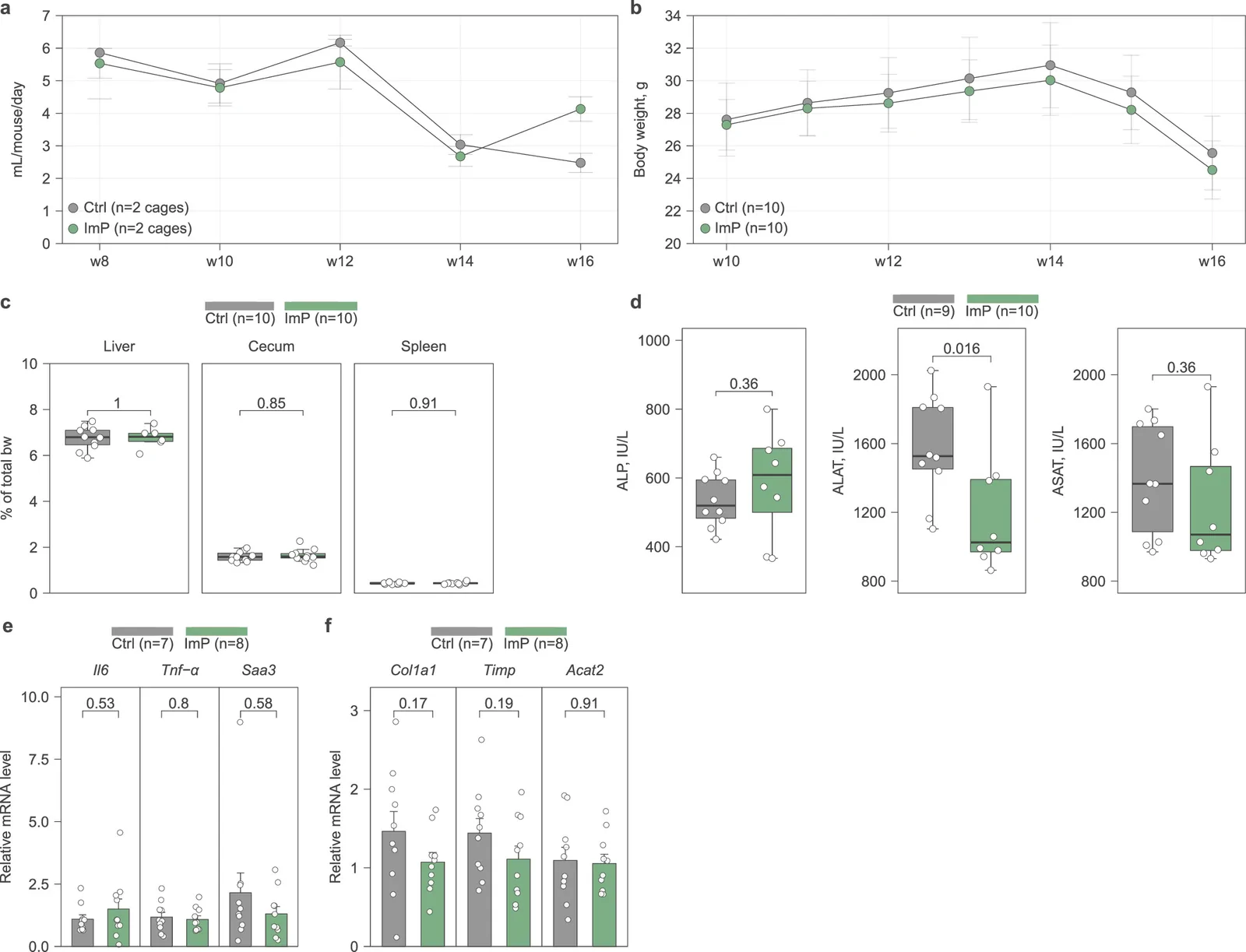
Anthropometric parameters, serum liver enzymes and gene expression levels in liver of wild type mice treated with and without imidazole propionate on a 0.1% 3,5-diethoxycarbonyl-1,4-dihydrocollidine (DDC) diet. **a-b**. Water intake and body weight in 8-week-old mice treated with vehicle (Ctrl) or imidazole propionate (ImP) (data are shown per cage) on a 6-week chow diet followed by 2 weeks on a DDC diet (*n =* 10 per group). **c**. Organ weights (as percentage of body weight) at sacrifice in mice treated with and without ImP. **d**. Serum levels of ALP, ASAT and ALAT at sacrifice. For panels **c**-**d**, box plot center lines indicates the median, the bounds of the box indicate the 25th and 75th percentiles, and the whiskers indicate the minimum and maximum values within 1.5 × the interquartile range. **e-f**. Relative mRNA levels of inflammation- and fibrosis-related genes in mouse liver extracts. Bars represent the mean, and error bars represent the standard error of the mean (SEM). P-values were calculated using two-sided Mann-Whitney *U*-tests. ALP: alkaline phosphatase, ASAT: aspartate aminotransferase, ALAT: Alanine aminotransferase, Il6: interleukin 6, Saa3: serum amyloid 3, Tnfα: tumor necrosis factor alpha, Acat2: Acetyl-CoA acetyltransferase, Col1a1: alpha-1 type I collagen, Timp: TIMP metallopeptidase inhibitor 1, WAT: white adipose tissue. Source data

Next, we investigated the effect of ImP administration in a diet-induced model of PSC^23^ by supplementing mice on chow diet with 0.1% 3,5-diethoxycarbonyl-1,4-dihydrocollidine (DDC) for 2 weeks (Fig. 5e). Plasma concentrations of both ImP and urocanate increased in ImP-treated mice compared with Ctrl mice (Fig. 5f). There were no significant differences in water intake, body weight or organ weights of the mice by treatment group (Extended Data Fig. 7a–c). As expected, administration of DDC led to an overall increase in portal inflammation, liver fibrosis and ductular reaction compared to Ctrl mice. However, despite the potency of DDC, liver fibrosis, portal inflammation and ductular reaction were more prominent in ImP-treated compared to Ctrl mice (Fig. 5g,h). Serum ALP as well as ASAT levels were comparable between groups, with a small significant reduction of ASAT levels in ImP-treated mice compared to Ctrl. There were no differences in the expression of hepatic pro-inflammatory and pro-fibrotic genes between the ImP and Ctrl groups (Extended Data Fig. 7e,f), again possibly a result of ImP primarily affecting portal areas (Supplementary Fig. 3).

### ImP activates mTORC1 in cholangiocytes

**Fig. 6:**
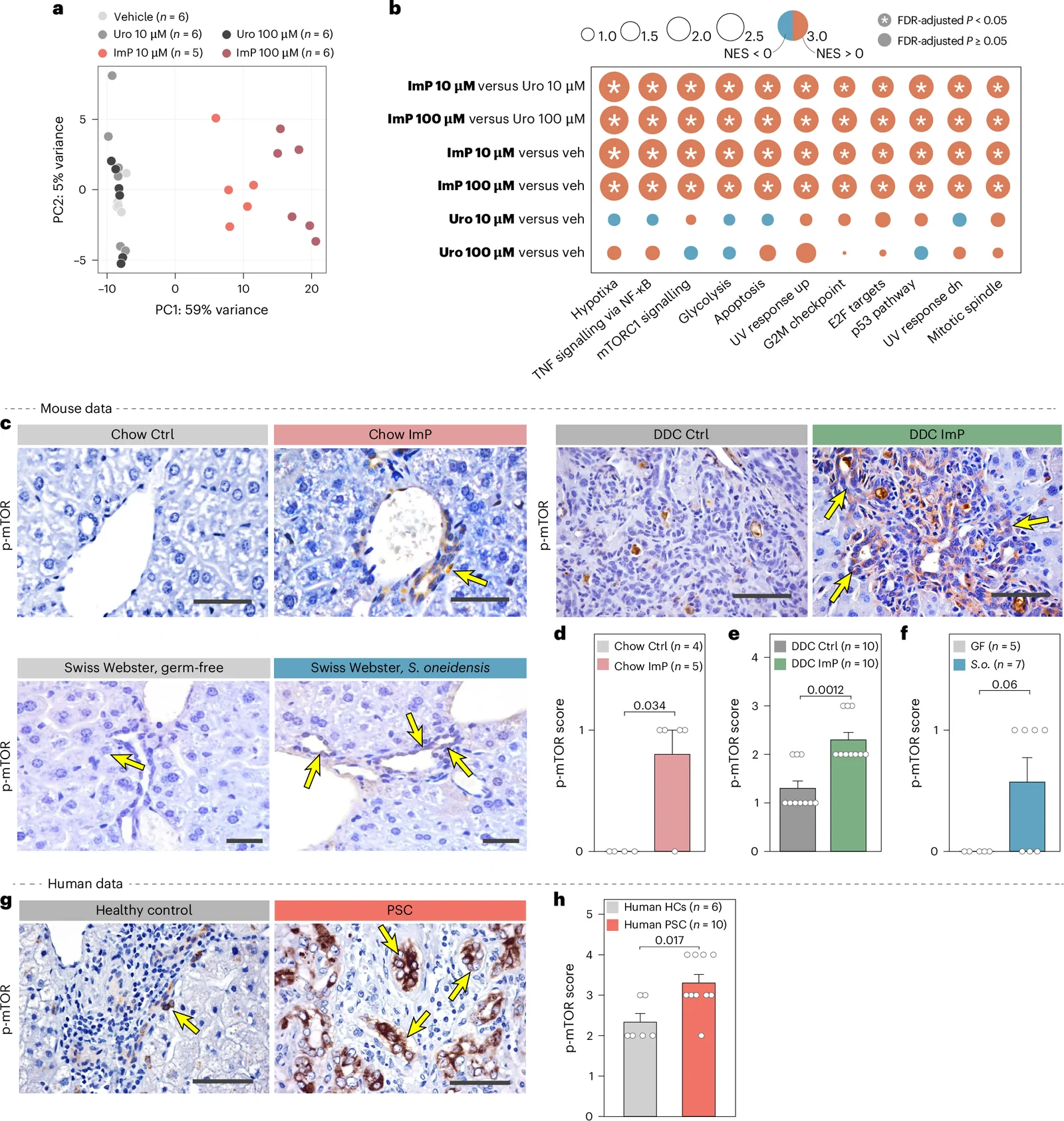
ImP activates the mTORC1 pathway in cholangiocytes. **a**, Principal component analysis of normalized RNA sequencing data from H69 cholangiocytes stimulated with ImP or urocanate at 10 µM and 100 µM. The first two principal components (PCs) are shown. One sample from the 10 µM ImP group was excluded as a clear outlier. The experiment was performed in three independent biological replicates, each with two technical replicates. **b**, Gene set enrichment analysis (GSEA) of Hallmark gene sets across DESeq2 comparisons (non-bold text indicates reference categories). Enrichment *P* values were calculated using two-sided tests (using fgsea::fgsea()) and adjusted for multiple comparisons using the FDR. Gene sets with an FDR-adjusted *P* < 1 × 10^−6^ from the GSEA in the ImP 100 µM versus Uro 100 µM comparison are shown. NES, normalized enrichment score. **c**–**f**, Representative liver sections stained for p-mTOR (**c**) and quantification of p-mTOR^+^ cells in mice fed chow (*n* = 4 and 5 per group) (**d**) or DDC diet (*n* = 10 per group) (**e**), vehicle-treated controls (Ctrl) or ImP-treated, germ-free (GF) and *S.* *oneidensis* (*S.o*.)-colonized mice (*n* = 5 and 7 per group) (**f**). **g**,**h**, Representative liver sections stained for p-mTOR (**g**) and quantification of p-mTOR^+^ cells in liver samples from HCs (*n* = 6) and individuals with PSC (*n* = 10) (**h**). Arrows indicate p-mTOR^+^ biliary epithelial cells (*n* = 6 and 10 per group). For **d**, **e**, **f** and **h**, bars represent the mean values; error bars, s.e.m. Statistical significance for individual comparisons was tested using two-sided Mann–Whitney *U*-tests. Scale bars, 50 μm. Source data

**Extended Data Fig. 8:**
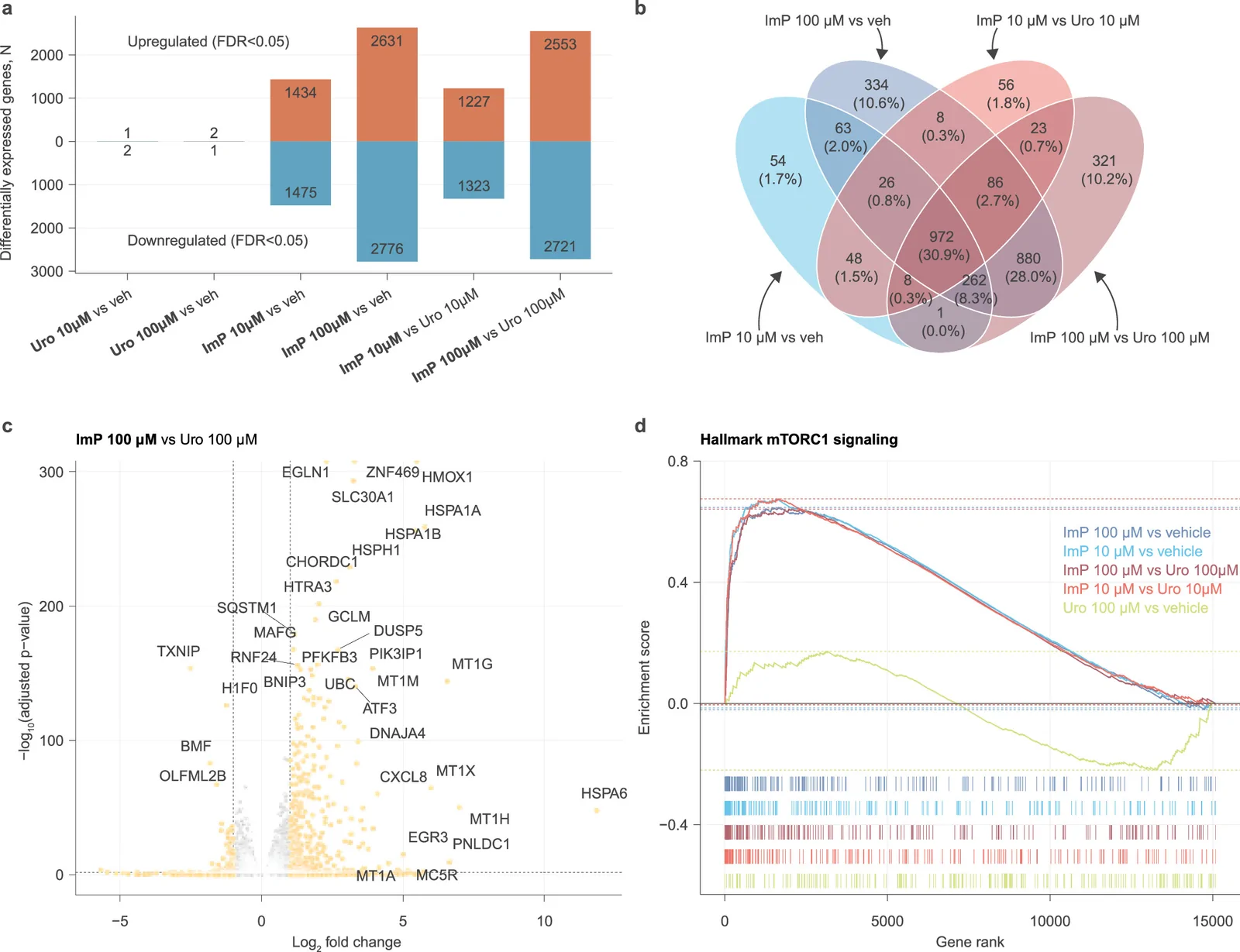
Imidazole propionate-driven activation of mTORC signaling-related genes in human cholangiocyte H69 cells. **a**. Shows the number of upregulated and downregulated genes from differential expression analyses using different comparisons, based on DESeq2 two-sided Wald test Benjamini-Hochberg false discovery rate (FDR) adjustment (FDR-adjusted p-value < 0.05). **b**. Venn diagram of overlapping and unique differentially expressed genes (DESeq2 two-sided Wald tests with FDR-adjusted p-value < 0.05). **c**. Volcano plot illustrating the relationship between individual gene log_2_(fold changes) and -log_10_(FDR)-adjusted p-values, estimated using DESeq with log fold change shrinkage (lfcShrink), in H69 cells treated with 100 µM imidazole propionate (ImP) compared to 100 µM urocanate (uro). Genes with an adjusted p-value < 1e-06 and an absolute log_2_(fold change) > 3 are labeled. **d**. Enrichment plot of the Hallmark mTORC1 signaling pathway genes for various comparisons, with rug plots showing each gene’s individual rank (based on t-statistics from the DESeq analyses). veh = vehicle. Source data

To identify molecular targets of ImP in cholangiocytes, we performed RNA sequencing of ImP-treated human cholangiocyte H69 cells. We observed a significant dose-dependent impact on the transcriptome, illustrated by enrichment of gene sets annotated to hypoxia, tumour necrosis factor signalling by nuclear factor kappa-light-chain-enhancer of activated B cells and mTORC1 signalling, while no such effects were observed in urocanate-treated cells (Fig. 6a,b and Extended Data Fig. 8). These findings were consistent with our previous observation in hepatocytes that short-term ImP induces mTORC1 pathway activity by promoting the upstream p38γ/δ activity^13^. To assess whether this mechanism was also active in vivo, we stained livers of mice treated with ImP with an antibody against phosphorylated (active) mTOR (p-mTOR). ImP treatment increased the number of p-mTOR^+^ cholangiocytes in mice fed either standard chow or chow supplemented with DDC (Fig. 6c–f). A similar trend was observed in *S.* *oneidensis-*colonized mice (Fig. 6c,e). To determine whether mTOR signalling is similarly induced in human disease, we performed p-mTOR staining on liver sections from individuals with PSC and HCs. We observed increased p-mTOR staining in PSC samples compared to HCs (Fig. 6g,h), consistent with the possibility that disease-associated factors like ImP contribute to mTOR activation in PSC. In line with a role of mTOR activation in PSC, our data suggest that mTOR inhibition may be protective: in a cohort of *n* = 182 people who received LT for PSC (Supplementary Table 15), exposure to the mTOR inhibitor sirolimus at or before the 1 year follow-up visit was less common among those who subsequently developed rPSC (*n* = 7, 6%) than among those who did not (*n* = 11, 16%; *P* = 0.04).

### Genetic ablation of p38γ/δ abrogates ImP-induced biliary inflammation and fibrosis

**Fig. 7:**
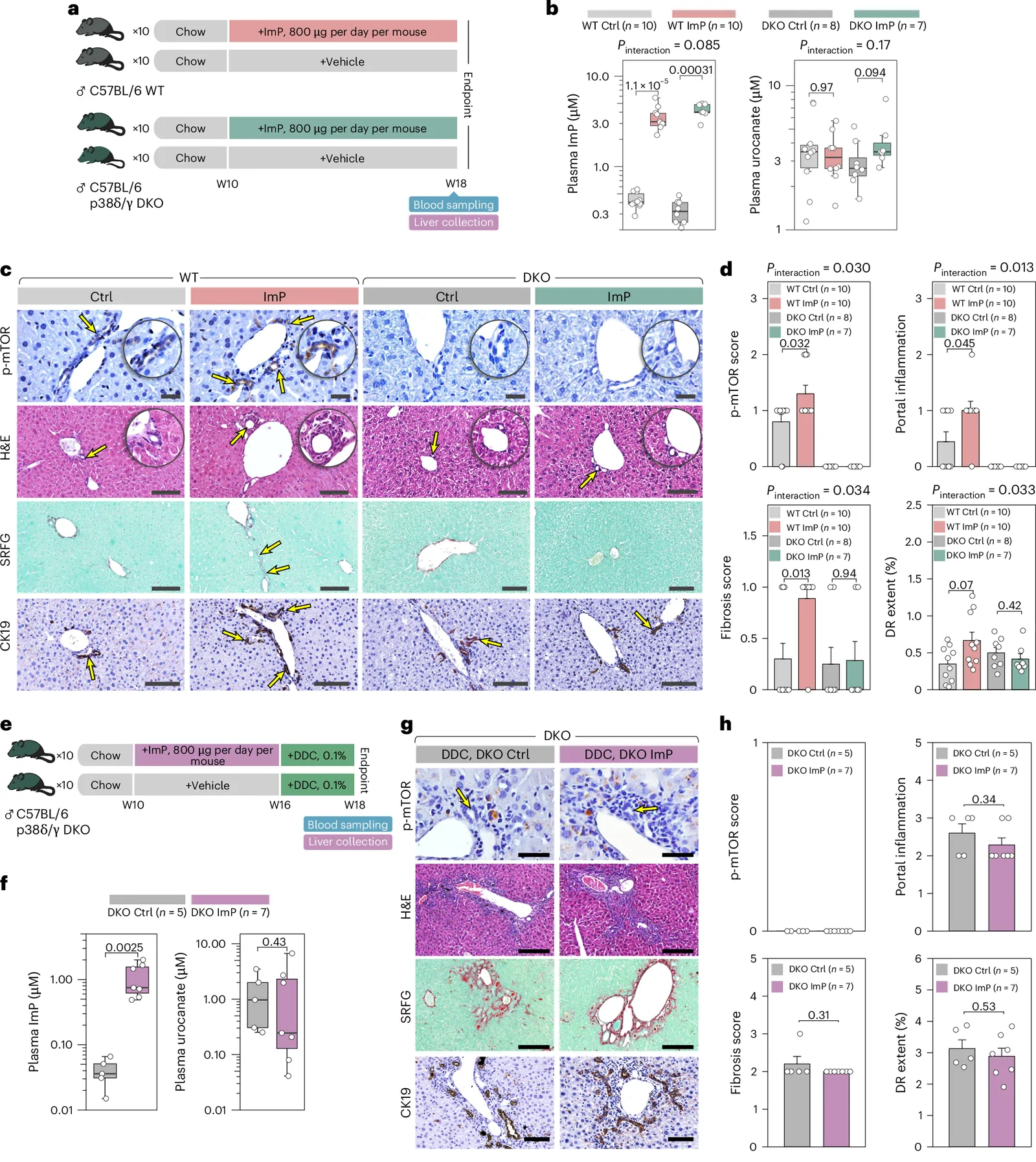
p38γ/δ is necessary for ImP-induced biliary inflammation and fibrosis. **a**, Schematic representation of the experimental design using C57BL/6J WT mice and p38γ/δ whole-body DKO mice on a chow diet (*n* = 7 and 10 mice per group). **b**, Plasma levels of ImP and urocanate in WT and DKO mice treated with vehicle (Ctrl) or ImP for 8 weeks on a chow diet. **c**,**d**, Representative images (**c**) and relative quantification (**d**) of H&E, SRFG and immunohistochemistry for CK19 in livers of WT and DKO mice treated without or with ImP on a chow diet. Scale bars, 100 μm (H&E, SRFG) and 50 μm (CK19). Arrows indicate areas of portal inflammation (magnified in the circles; H&E), collagen fibres (SRFG) or ductular reaction (CK19). **e**, Schematic representation of the experimental design using DKO mice on a 0.1% DDC diet (*n* = 5 and 7 per group). **f**, Plasma levels of ImP and urocanate in DKO mice treated with vehicle (Ctrl) or ImP for 8 weeks, on a 6-week chow diet followed by 2 weeks on a DDC diet. **g**,**h**, Representative images (**g**) and relative quantification (**h**) for H&E, SRFG and immunohistochemistry for CK19 in livers of mice treated with or without ImP on a chow diet. Scale bars, 100 μm (H&E, SRFG) and 50 μm (CK19). Arrows indicate areas of portal inflammation (H&E), collagen fibres (SRFG) or ductular reaction (CK19). For **b** and **f**, box plot centre lines indicate the median, the bounds of the box indicate the 25th and 75th percentiles and the whiskers indicate the minimum and maximum values within 1.5× the IQR. For **d** and **h**, bars represent the mean values; error bars, s.e.m. *P* values for two-group comparisons were calculated using two-sided Mann–Whitney *U*-tests. For comparisons involving genotype and treatment, *P* values were derived from factorial analyses including genotype, treatment and their interaction. Source data

**Extended Data Fig. 9:**
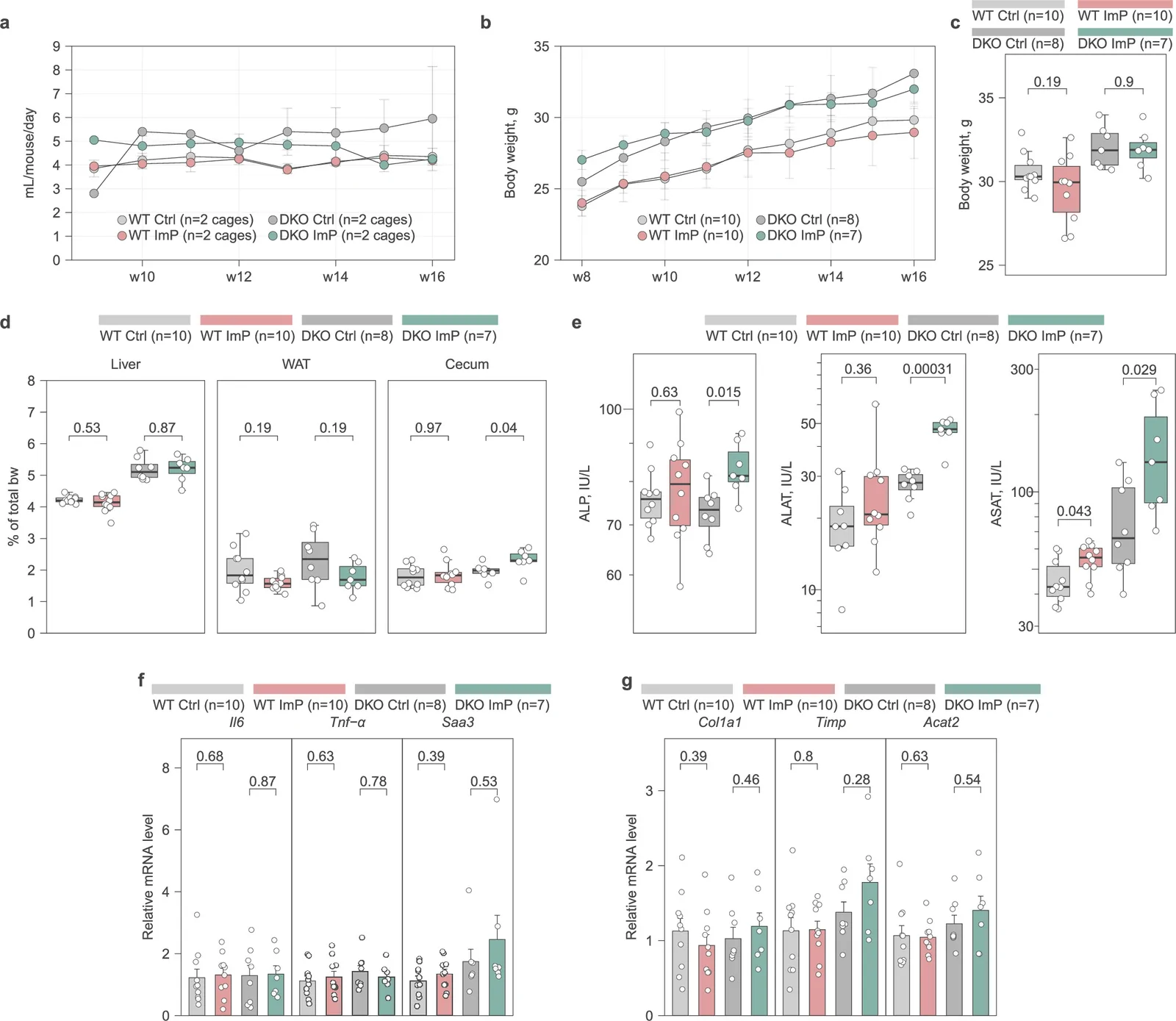
Anthropometric parameters, serum liver enzymes and gene expression levels in the liver of wild type (WT) and p38γ-δ knock out mice (DKO) treated with and without imidazole propionate on a chow diet. **a-b**. Water intake and body weight in 8-week-old WT and DKO mice treated with vehicle (Ctrl) or imidazole propionate (ImP) on a chow diet (*n =* 7 and 10 per group with data shown per cage). **c**. Body weights at time of sacrifice. **d**. Organ weights (as percentage of body weight) at sacrifice in mice treated with and without ImP. **e**. Serum levels of ALP, ASAT and ALAT at sacrifice. For panels **c**-**e**, box plot center lines indicate the median, the bounds of the box indicate the 25th and 75th percentiles, and the whiskers indicate the minimum and maximum values within 1.5 × the interquartile range. **f-g**. Relative mRNA levels of inflammation- and fibrosis-related genes in mouse liver extracts. Bars represent the mean, and error bars represent the standard error of the mean (SEM). P-values were calculated using two-sided Mann-Whitney *U-*tests. ALP: alkaline phosphatase, ASAT: aspartate aminotransferase, ALAT: Alanine aminotransferase, Il6: interleukin 6, Saa3: serum amyloid 3, Tnfα: tumor necrosis factor alpha, Acat2: Acetyl-CoA acetyltransferase, Col1a1: alpha-1 type I collagen, Timp: TIMP metallopeptidase inhibitor 1, WAT: white adipose tissue. Source data

We have previously demonstrated that ImP can induce mTORC1 signalling through p38γ and, to a lesser extent, p38δ (refs. ^13,24^). To investigate whether this pathway mediates ImP-induced biliary inflammation and liver fibrosis in vivo, we generated mice with a whole-body deletion of p38γ/δ (double knockout, DKO) (Supplementary Fig. 4) and administered ImP in the drinking water to both 6-week-old wild-type and DKO mice for 8 weeks (Fig. 7a). As expected, ImP treatment increased circulating ImP but not urocanate to similar levels in both the DKO mice and wild-type littermate controls (Fig. 7b). Neither ImP treatment nor genotype affected food intake, body weight or organ weights (Extended Data Fig. 9a–d). As expected, ImP administration to wild-type mice increased liver p-mTOR staining, portal inflammation, liver fibrosis and ductular reaction compared to wild-type control mice (Fig. 7c,d). By contrast, p-mTOR staining was abolished and ImP did not induce portal inflammation, liver fibrosis or ductular reaction in DKO mice (Fig. 7c,d). Serum ALP, ASAT and ALAT were increased in DKO mice treated with ImP compared to Ctrl mice (Extended Data Fig. 9e). This finding may suggest that ImP had additional effects on the liver not mediated by mTORC1. There were no differences in the expression of hepatic inflammatory and pro-fibrotic genes in ImP-treated or Ctrl groups (Extended Data Fig. 9f, g).

**Extended Data Fig. 10:**
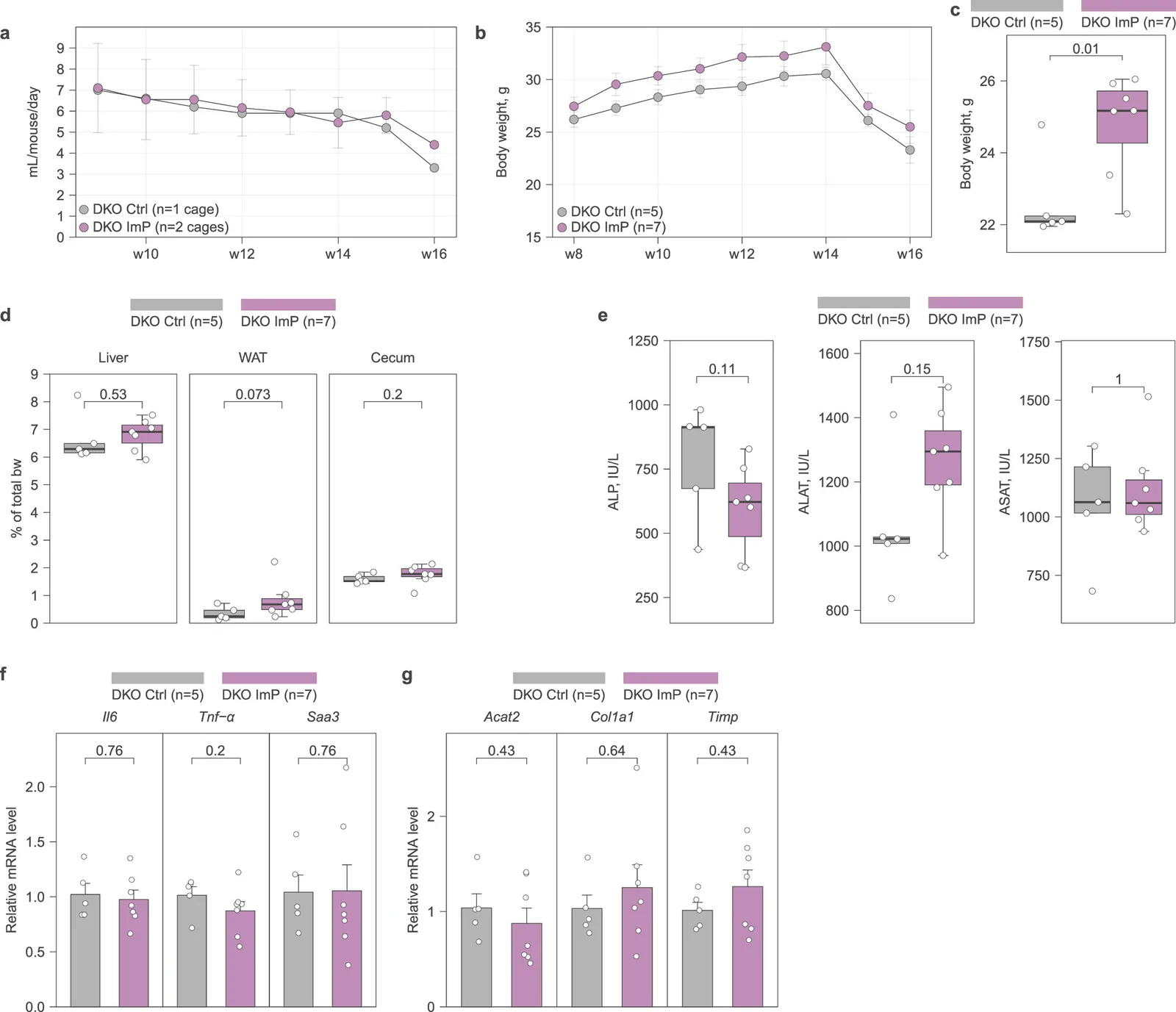
Anthropometric parameters, serum liver enzymes and liver gene expression levels in p38γ-δ knock out (DKO) mice treated with and without imidazole propionate on a 0.1% 3,5-diethoxycarbonyl-1,4-dihydrocollidine (DDC) diet. **a-b**. Water intake and body weight in DKO 8-week-old mice treated with vehicle (Ctrl) or imidazole propionate (ImP) (data are shown per cage) on a 6-week chow diet followed by two weeks on a DDC diet (*n =* 10 per group). **c**. Body weights at time of sacrifice. **d**. Organ weights (as percentage of body weight) at sacrifice in mice treated with or without ImP. **e**. Serum levels of ALP, ASAT and ALAT at sacrifice. For panels **c**-**e**, box plot center lines indicate the median, the bounds of the box indicate the 25th and 75th percentiles, and the whiskers indicate the minimum and maximum values within 1.5 × the interquartile range. **f-g**. Relative mRNA levels of inflammation and fibrosis related genes in mouse liver extracts. Bars represent the mean, and error bars represent the standard error of the mean (SEM). P-values were calculated using two-sided Mann-Whitney *U*-tests. ALP: alkaline phosphatase, ASAT: aspartate aminotransferase, ALAT: Alanine aminotransferase, Il6: interleukin 6, Saa3: serum amyloid 3, Tnfα: tumor necrosis factor alpha, Acat2: Acetyl-CoA acetyltransferase, Col1a1: alpha-1 type I collagen, Timp: TIMP metallopeptidase inhibitor 1, WAT: white adipose tissue. Source data

When the DKO mice were challenged with a 2-week DDC diet (Fig. 7e,f and Extended Data Fig. 10), we did not observe any effect of ImP treatment on mTORC1 activation or worsening of fibrosis, inflammation or ductular reaction (Fig. 7g,h), showing that ImP-driven biliary injury in mice was dependent on p38γ/δ and thus functional mTORC1 signalling. There were no differences in the expression of hepatic pro-inflammatory and pro-fibrotic genes in ImP-treated or Ctrl groups (Extended Data Fig. 10f, g).

## Discussion

We identified the bacterial histidine metabolite ImP as a driver of PSC by alternative p38 activation. Our findings represent a molecular mechanism linking the gut alterations and liver manifestations in PSC, highlighting pathogenetic principles that are relevant for other diseases involving the gut–liver axis.

We demonstrated broad metabolic alterations in PSC, but a strict filtering pipeline to detect potential disease drivers enabled the identification of circulating ImP as being consistently elevated in PSC cohorts compared with healthy individuals, as well as when considering non-advanced disease and other metabolic or cholestatic liver diseases. Our findings are consistent with prior untargeted metabolomics data in which ImP was reported among metabolites elevated in PSC compared with HCs^25^, as well as earlier observations of increased urinary ImP excretion in individuals with gastrointestinal diseases^25^. However, ImP has not been specifically investigated in PSC.

Mechanistically, we identified the alternative p38 signalling through mTORC as a molecular target for cholangiocyte pro-fibrotic and inflammatory phenotypes. This finding is consistent with a previous study that demonstrated that the mTORC1 pathway was upregulated in bile ducts in individuals with PSC^26^. mTORC has been shown to drive cholangiocyte inflammation, proliferation and fibrosis, both in chronic liver diseases and in cholangiocarcinoma^26,27^, which can develop in PSC. Our data showed that deletion of p38γ/δ prevents ImP-driven inflammation and fibrosis, indicating that targeting p38 MAPK or mTORC directly could be efficacious in PSC. Although DKO models provide mechanistic support, they may also introduce off-target effects and should therefore clearly be interpreted with caution. However, pirfenidone (a p38 inhibitor with antifibrotic effect) has been shown to reduce cholestatic liver injury in a chemically induced mouse model of cholestasis^28^. Furthermore, in a small pilot study of pirfenidone in PSC, a trend towards reduced histological fibrosis was observed, although the drug was poorly tolerated^29^. The associations observed between ImP and later rPSC, and less exposure to sirolimus in individuals with later recurrence, also provide a rationale for further testing of pharmacological inhibition of these pathways; for example, using mTOR inhibitors.

ImP is a marker of microbial ecosystems with low diversity^12^, such as in IBD^30,31^. Individuals with IBD without PSC exhibited elevated ImP levels compared with controls, although these were lower than in PSC, and ImP levels were similar in PSC irrespective of concurrent IBD. ImP was only modestly elevated in PBC and MASLD, collectively suggesting a general increase in microbial ImP production across disease-associated gut environments, with the highest levels observed in PSC. Although ImP levels converge across liver diseases of different aetiologies at the time of LT, they are disproportionately elevated in PSC earlier in the disease course, supporting a PSC-specific component driving ImP production. This interpretation is further supported by the persistent elevation of ImP after LT for PSC, suggesting that increased ImP is not simply a consequence of impaired liver function but may instead reflect ongoing dysbiosis^7^. Despite these signs of specificity, high ImP has been observed in, for example, type 2 diabetes and cardiovascular disease^13,14,17,32–34^, which indicates that ImP may be relevant in several conditions in which the mTORC pathway is involved. There is limited overlap between risk of PSC and, for example, cardiovascular disease^35^, suggesting that there are other determinants of ImP-driven disease specificity, and the role and effect of ImP in any individual is a consequence of multiple factors like genetic susceptibility, age, sex and other exposures or risk factors.

ImP is produced by gut bacterial enzymes encoded by the *urdA* gene; however, accurate quantification at the DNA level is challenging owing to substantial sequence heterogeneity among *urdA*-encoding genes^13^. Circulating ImP levels have been repeatedly associated with an overlapping set of specific bacteria in other metabolic conditions^14,17,36^. Here, we showed that bacteria that have been previously reported to be increased in PSC, such as *E.* *clostridioformis* and *E.* *boltae*^10^, or that are known to be associated with ImP-circulating levels, such as *R.* *gnavus*^17^, are associated with high circulating levels of ImP and with low bacterial diversity. Together with the lack of normalization of gut microbiota composition after LT for PSC^7^ and the known impact of advanced liver disease on promoting dysbiosis and expansion of pathobionts^19^, these observations suggest that the intestinal environment in PSC predisposes to increased ImP production. As disease progresses towards cirrhosis, non-PSC-specific factors related to advanced liver disease may then become the dominant determinants of circulating ImP.

Experimental data in mice have shown that broad-spectrum antibiotics reduce microbial ImP production^37^, and we show here that colonization with an urdA-expressing strain, combined with dietary supplementation with urocanate, increased systemic ImP levels and signs of biliary injury. Together, these findings form a mechanistic link between gut microbial ImP biosynthesis and a biliary phenotype, and principally suggest that targeting the gut or gut microbes may be a therapeutic avenue. In humans, however, broad-spectrum antibiotics are unlikely to constitute a viable strategy and may paradoxically increase ImP levels by favouring a low-diversity, ImP-producing microbial ecosystem^37^. In addition, such interventions may further impair microbiome integrity and promote antimicrobial resistance. Nonetheless, our finding that ImP was particularly low in proctocolectomized individuals with ileostomy could point towards a clinical benefit of intervention-based restriction of gut ImP production, and is consistent with prior data demonstrating better clinical outcomes in PSC after proctocolectomy with ileostomy^3^. Potential translational strategies may include more targeted approaches, such as limiting access to the dietary ImP substrates histidine and urocanate and reducing the capacity of ImP-producing strains to produce ImP through chronic UrdA inhibition^37,38^.

PSC typically, over decades of progressive bile duct injury, causes liver failure and death^39^. The exact cause of PSC is unclear but involves a complex interaction of genetic, environmental and immune factors. The impact of ImP in experimental models in vivo was mainly seen on liver histology and must be considered modest. In line with the natural history, we speculate that continuous exposure to elevated circulating levels of ImP over time, together with other factors, contributes to sclerosis of the bile ducts and disease progression. The physiological relevance of compound concentrations used in experimental science is also an important discussion point. We speculate that the long exposure time in humans could, in part, also explain the modest effect of short-term high ImP concentration in the in vivo models compared with the potent disease associations observed with lower serum ImP concentrations in humans. Notably, in the colonization model with an urdA-expressing strain, circulating ImP concentrations were within the upper range of what we find in human PSC, suggesting that these concentrations may be sufficient to induce biliary injury in vivo. The single-timepoint cross-sectional serum ImP concentrations in PSC varied from 2 nM to 500 nM, suggesting that the gut microbial potential to produce ImP—and hence its clinical impact—varies considerably between individuals. This is illustrated by the strongest effect on PSC survival seen in the upper tertile of ImP levels across all studied populations, suggesting that this is a subgroup in which ImP-directed therapies could be especially relevant.

Importantly, ImP may reach the cholangiocyte in portal blood directly perfusing large bile ducts^40^, by systemic (arterial) circulation or even from the bile duct, as ImP is present in human bile^41^, in line with its presence in mouse liver tissue, gallbladder tissue and bile shown here. Indeed, exposing cholangiocytes to ImP led to a major transcriptomic rewiring and increased secretion of pro-inflammatory and pro-fibrogenic factors, thus resembling activated cholangiocytes responding to inflammation. Although the exact concentrations of ImP used in in vitro systems are high and not directly translatable to in vivo conditions, the experiments provide proof of principle of mechanisms underlying the effects observed in vivo. The recent discovery of the imidazoline 1 receptor as a probable target of ImP could lead to further molecular characterization of the ImP–mTORC pathway and potentially new therapeutic opportunities. As ImP may also influence macrophages and other cells expressing its receptor^34^, and given the autoimmune features of PSC, studies of the impact of ImP on, for example, different immune cell subsets should also be a priority.

At present, there is no cure for PSC except LT for advanced cases, after which the disease often recurs. Detailed knowledge of disease mechanisms is a prerequisite for the development of an effective treatment. We have identified ImP as a circulating metabolite associated with PSC transplant-free survival. By demonstrating that ImP can activate cholangiocytes ex vivo and in vivo and elucidating the molecular pathways involved, we highlight that the development of treatments that can reduce production of ImP or inhibit ImP-induced cellular signalling may represent an important step forward in this orphan disease.

## Methods

### Participants

#### Inclusion, ethics and consent

Unless stated otherwise, there were no explicit clinical exclusion criteria, and sample availability determined sample sizes. All study subjects gave informed, written consent. The participants were not given any compensation. The study protocol conformed to the ethical guidelines of the 1975 Declaration of Helsinki and was approved by the relevant institutional review boards (IRBs) and ethics committees in each participating country: Norway (2010/1540, 2011/2572, 2015/2140, 2012/286, 2016/1690 and 568878); Sweden (Dnr 571-18 and 2018/1111-32); Italy (PBC322 numbers 2969 and 5129/541-1); the USA (Mayo Clinic IRB for PSC studies, IRB numbers 670-02 and 16-005892); and Denmark (Central Denmark Region Ethics Committee, 1-10-72-146-16).

#### Untargeted discovery cohort

The discovery cohort consisted of cross-sectionally collected EDTA plasma samples from 132 liver pre-LT and 29 post-LT patients with PSC (5 year blood sample, no rPSC diagnosis made at time of sampling) who were prospectively recruited between 2008 and 2015 at the tertiary care hospital Oslo University Hospital, Rikshospitalet (Oslo, Norway). Thirty-three age-matched healthy donors from the Norwegian Bone Marrow Donor Registry were also included. Ethical approval was obtained from the Regional Committee for Medical and Health Research Ethics in South-Eastern Norway (2011/2572, 2015/2140 and 2016/1690), which covers all analyses of Norwegian patients with PSC before or after transplant as well as HCs (that is, NO-1, NO-2 and the longitudinal PSC cohort; additional cohorts listed below).

#### Expanded targeted cohort (NO-1)

An expanded, cross-sectional, technical validation cohort consisted of 164 individuals with pre-LT PSC and 100 age-matched HCs attending Rikshospitalet. Of these, 117 individuals with PSC and 29 HCs temporally overlapped with the discovery cohort (that is, samples were taken from the same blood draw). Both plasma and serum were analysed in parallel.

#### Independent targeted cohort (NO-2)

An independent validation cohort from Rikshospitalet consisted of serum from 151 individuals with pre-LT PSC who had blood sampled cross-sectionally.

#### External validation cohort (SE)

An external validation cohort consisted of 139 individuals with pre-LT PSC who were recruited at Karolinska University Hospital (Stockholm, Sweden) between 2008 and 2012, as well as 51 healthy donors recruited at Sahlgrenska University Hospital (Gothenburg, Sweden). All participants had donated one serum sample. Ethical approval: Dnr: 571-18 and 2018/1111-32.

#### External validation cohort (US)

An external validation cohort from the USA (*n* = 609) was obtained from the PSC Scientific Community Resource^42^. Ethical approval was obtained from the Mayo Clinic (IRB numbers 670-02 and 16-005892).

#### Longitudinal PSC cohort

A longitudinal Norwegian cohort consisted of 158 individuals with PSC who underwent LT at Rikshospitalet between 1990 and 2016 (ref. ^16^) and for whom EDTA plasma samples were available. Individuals in this cohort were sampled both directly before and serially after (3 months, 1, 2, 3, 5 and 10 years) their first LT (totalling 567 plasma samples; median four samples donated; range, 1–7). In total, 51 participants overlapped with the expanded technical validation cohort, albeit with temporally non-matched plasma samples. Individuals included in the final analyses (see outcomes and definitions below) are described in Supplementary Table 9. Details regarding clinical definitions in the post-transplant setting are described in the Supplementary Note.

For the study of the effect of sirolimus on later rPSC, adults who underwent LT for PSC between 1988 and 2017 and who were evaluated at a 1 year follow-up were included (Supplementary Table 15).

#### PBC cohorts

A cross-sectional cohort comprised 71 consecutive individuals with PBC and 26 HCs enrolled at Bicocca University Hospital, Milan, and Policlinico Umberto I, Rome, for whom serum samples were available for ImP measurement. Ethical approval was obtained in Milan, Italy (PBC322; n.2969), and Rome, Italy (n.5129; 541/1).

A cross-sectional cohort comprised 236 individuals with either incident (*n* = 79) or prevalent (*n* = 157) PBC, from whom serum samples were obtained at Aarhus University Hospital and Aalborg University Hospital, Denmark^43^. The study was approved by the ethical committee of the Central Denmark Region (1-10-72-146-16).

#### IBD cohort

A total of 51 serum samples were obtained from the prospective, population-based IBD cohort (IBSEN^16,44^). The samples were drawn from a set of samples collected 20 years after IBD diagnosis from individuals who had no evidence of PSC as evaluated by MR cholangiopancreatography at the time of blood collection. Ethical approval was obtained from the Regional Committee for Medical and Health Research Ethics in South-Eastern Norway (2010/1540).

#### MASLD cohort

The cross-sectional cohort comprised 71 adults with MASLD (42 males (59.2%); median age, 52 years (IQR, 23)) who were referred for liver elastography from gastroenterology, diabetes and morbid obesity outpatient clinics and consecutively enrolled at Haraldsplass Deaconess Hospital, Bergen, Norway, between 2023 and 2025. Ethical approval was obtained from the Regional Committee for Medical and Health Research Ethics in Western Norway (number 568878).

All participants met criteria for a MASLD-compatible diagnosis, defined by hepatic steatosis, absence of clinically significant alcohol intake (>12 units per week for males or >6 units per week for women) and exclusion of alternative or concomitant liver disease. Participants had a mean BMI of 35.6 ± 6.5 (s.d.), and 70 (98.6%) had at least one metabolic trait. Overall, 69 participants (97.2%) had overweight or obesity (BMI ≥ 25 kg m^2^), including 62 (87.3%) with obesity (BMI ≥ 30 kg m^2^); 30 (42.3%) had hypertension, 23 (32.4%) had hypercholesterolaemia and 21 (29.6%) had type 2 diabetes. Individuals receiving systemic corticosteroids or other immunosuppressive agents within 12 weeks before study inclusion were not eligible. All participants underwent standardized B-mode ultrasound examination on the day of serum or plasma sampling to confirm hepatic steatosis, defined as diffusely hyperechogenic liver parenchyma compared with right kidney parenchyma, followed by liver stiffness assessment in the same session^45^.

#### Transplant cohort

For analysis of ImP levels in advanced-stage liver disease, serum samples obtained at LT evaluation were selected from 39 individuals with PSC, 21 with PBC and 13 with alcohol-related liver disease who subsequently underwent LT. Individuals with low-grade or high-grade dysplasia as the indication for transplantation were excluded. Samples were obtained within 1 year before transplantation.

#### Human PSC liver samples

For immunohistochemical analysis of human PSC liver tissues, formalin-fixed liver explant tissue was obtained from ten individuals who underwent LT for PSC in Oslo, Norway, between 2004 and 2012. All patients provided written informed consent. Median age at transplant was 37 years (range, 27–61 years). At the time of transplantation, median (range) biochemical parameters were 20 (4–219) µmol l^−1^ for bilirubin, 41 (22–49) g l^−1^ for albumin, 86 (20–258) U l^−1^ for AST, 129 (44–290) U l^−1^ for ALT, 250 (73–859) U l^−1^ for ALP and 1.0 (0.9–2.4) for INR. No tissue is available for additional analyses. Ethical approval was obtained from the Regional Committee for Medical and Health Research Ethics in South-Eastern Norway (2012/286).

#### Human control liver samples

As histological controls, human liver samples with normal histology (*n* = 6) were obtained from organ donors at the ‘Paride Stefanini’ Department of General Surgery and Organ Transplantation, Sapienza University of Rome, Italy. No donor organs were obtained from executed prisoners or other institutionalized individuals. Ethical approval was obtained in Rome, Italy (541/1).

#### Sex and gender reporting

Sex was incorporated into the study design and was determined from population records. Gender was not assessed. Adjustment for sex in the initial analyses of ImP levels did not materially influence the observed associations, and therefore no further or post hoc analyses specifically examining sex-dependent effects were performed, given that there was no a priori hypothesis related to sex-specific effects.

Sex was also considered during the design of the animal experiments. In accordance with the principles of the 3Rs (replacement, reduction and refinement), we chose to perform the study using a single sex to minimize animal use. Only male mice were included because PSC shows a clear male predominance in humans, making male animals the most clinically relevant initial choice.

#### Outcomes, definitions and diagnostic criteria

PSC was defined according to accepted criteria^1^. For the cross-sectional cohorts, the composite endpoint event was LT or death. LT was restricted to transplantations performed for PSC, irrespective of clinical or symptomatic burden. LT-free survival was calculated as the time from sampling to endpoint. For these analyses, subjects who donated blood, presumably in conjunction with a referral for LT (*n* = 25), or who had hepatobiliary cancer at the time of sampling (*n* = 13) were excluded.

### Untargeted metabolomics using ultra-high performance LC–MS/MS

A total of 193 plasma samples were analysed by ultra-high performance liquid chromatography with tandem mass spectrometry (UPLC–MS/MS) at Metabolon Inc. Metabolites were identified by comparison with library entries for purified standards or recurrent unknowns, by matching retention time/index, mass-to-charge ratio and chromatographic data. Of 1,265 detected metabolites, 243 were of unknown identity (termed ‘unclassified metabolites’). Further details are described in the Supplementary Note.

#### Targeted ImP and urocanate measurements

ImP and urocanate in serum samples from the external validation (SE) and PBC (Italy) cohorts were measured using targeted UPLC–MS/MS analysis as reported previously^13^, with minor modifications outlined in the Supplementary Note. For the NO-1, NO-2, US, longitudinal PSC, IBSEN IBD, PBC (DK) and MASLD (NO) cohorts, plasma and/or serum ImP was analysed using a panel for B-vitamins, kynurenines and a set of microbial metabolites at BEVITAL (Panel D, bevital.no) by LC–MS/MS as described in a previous publication^46^.

#### Stool shotgun metagenomics (USA)

##### Sampling protocol

Individuals with PSC from the USA were recruited through the PSC Scientific Community Resource^42^. Stool specimens were self-collected using additive-free mail-in kits, shipped overnight to the Mayo Clinic on ice packs and stored at −80 °C before downstream processing. Genomic DNA was subsequently isolated at the University of Minnesota Genomics Center using the Qiagen DNeasy 96 PowerSoil Pro QIAcube HT kit.

##### Sequencing and shotgun metagenomic read processing

Shotgun metagenomic sequencing was performed using an Illumina NovaSeq 6000 at the University of Minnesota Genomics Center with the Nextera library preparation kit. Sequencing generated 150 bp paired-end reads, with a mean sequencing depth of 9.16 million read pairs per sample. Raw reads underwent quality control and host DNA decontamination, followed by taxonomic classification using Kraken 2 (ref. ^47^) against the GTDB reference database (v.220)^48^, as previously described^49^.

##### Data analysis

To account for compositionality, species-level count data were transformed using the centred log-ratio transformation with the compositions::clr function and a pseudocount of 1. Before centred log-ratio transformation, we removed rare species by excluding taxa with zero counts in more than 80% of the samples.

#### In vivo experiments

Male wild-type C57BL/6J mice were purchased from Charles River Laboratories (Germany). Mice were maintained under a 12 h light cycle with access to food and water ad libitum. A chow diet (TD.130066, LabDiet) was used, enriched with 0.1% DDC (Merck; 137030-25G).

Mice harbouring loxP sequences flanking exon 3 p38γ (*Mapk12*) and exon 3 p38δ (*Mapk13*) were generated by Cyagen Biosciences (Supplementary Fig. 4). The strain was generated by homologous recombination in C57BL/6N embryonic stem cells, followed by microinjection into C57BL/6N blastocysts. The resulting offspring were crossed with C57BL/6NTac-*Gt(ROSA)26Sor*^*tm*16*Arte*^ Cre deleter mice to excise the neomycin selection cassette (Taconic Biosciences) and subsequently crossed with p38γ/δ^fl/fl^. Deletion of 95 bp and 127 bp from exon 3 of *Mapk12* and *Mapk13*, respectively, led to a frameshift resulting in a premature stop codon. No functional domain would be present on these putative proteins. All animal procedures were approved by the Gothenburg Animal Ethics Committee (Dnr 5.8.18-16056/2019).

Male mice (6-week-old C57BL/6J or p38γ/δ knockout) were randomized to receive either ImP (Santa Cruz, sc294276; 800 µg per day per mouse) or no ImP (Ctrl) in drinking water. Water and food consumption was determined twice weekly. After 8 weeks of treatment, all mice were killed, which was the defined endpoint for all mouse experiments, and body and organ weights were determined. Peripheral serum from the vena cava and liver tissue were collected and stored at −80°C for further analysis. Serum ASAT, ALAT and ALP were measured using Alinity c (Abbott Laboratories). Total RNA was isolated using the RNeasy kit with DNase treatment (Qiagen), and cDNA templates were generated using the high-capacity cDNA reverse transcription kit using random hexamers (Applied Biosystems). Reverse transcription qPCR assays were performed in 10 µl reactions with SYBR Green Master Mix buffer (Thermo Scientific). In a subgroup of C57BL/6J mice, blood was collected from the tail vein following vehicle or ImP treatment. Mice were then perfused with PBS through the inferior vena cava to remove blood before collecting organs. Gene expression data were normalized to the ribosomal protein L32. Primers were purchased from Sigma-Aldrich; sequences are listed in Supplementary Table 16.

Germ-free male Swiss Webster mice, 10–12 weeks old, were maintained in flexible film isolators under a strict 12 h light cycle. Germ-free status was verified regularly by anaerobic culturing in addition to PCR for the bacterial 16S rRNA gene. Experiments were performed on autoclaved chow diet (LabDiet) ad libitum. *S.* *oneidensis* MR-1 (ATCC 700550) was grown in Trypticase Soy Yeast Extract Medium (TSBYE) (Becton Dickinson) supplemented with 0.25% yeast under aerobic conditions at 29 °C. Bacteria were collected at a density of ~10^9^ cells per ml and administered to mice by oral gavage (200 µl per mouse) following a 4 h fasting period on day 0. Colonized mice were monitored closely in experimental isolators to ensure controlled conditions. Urocanate at a concentration of 500 µM in the drinking water was provided to colonized mice from week 3 onwards. The colonization experiment was performed on one batch of mice.

### Liver histology and immunohistochemistry

Details regarding liver histology and immunohistochemistry are described in the Supplementary Note.

### In vitro and ex vivo experiments

Details regarding in vitro experiments in H69 cells and ex vivo experiments in cholangiocyte organoids are described in the Supplementary Note.

### Statistical analyses

#### Handling of missing data

In our discovery (untargeted metabolomics) dataset, we detected a total of 1,265 metabolites, with 42,540 (17.3%) values missing. ImP was detected in all samples. There were no discernible differences in the distribution of missing values between the sample groups (Supplementary Fig. 5). We excluded metabolites missing in >50% of samples (157), of which 89% were annotated as drugs or unclassified. For the remaining 1,108 metabolites, missing values were imputed using multivariate imputation by chained equations predicted mean matching (MICE-pmm) method^50^ in the UnMetImp script in R, which specifically takes into account that xenobiotics are unlikely to be missing at random and should therefore have missing values imputed to zero. Age at sampling, leucocyte counts, platelet counts, bilirubin, albumin, ASAT, ALAT and ALP were included in the imputation. Missing data in the NO-1, NO-2 and SE cohorts were imputed for each cohort separately using the mice::mice function. Age and blood biochemical parameters were included in the imputation. ImP was quantified and detected in all samples. Missing data in the longitudinal cohort were not imputed given that only around half of the theoretical maximum of samples were collected and the mechanisms underlying the missing data could not be determined.

#### Rule-based feature selection pipeline (untargeted metabolomics cohort)

In the discovery cohort, we defined three rules to pinpoint putative disease-driven, gut microbiota-derived candidate metabolites in plasma samples. The candidate metabolite had to be (1) differentially abundant in PSC versus HCs in a multivariable binary logistic regression model that included age and sex as fixed covariates (FDR < 0.01); (2) associated with LT-free survival in a multivariable Cox proportional hazards model with sex and Mayo PSC risk score as fixed covariates (*P* < 0.05); and (3) the metabolite must show no evidence (*P* value threshold of 0.05) of returning to its corresponding level in HCs in PSC post-LT samples. To identify apparent metabolite candidates, we generated 40 imputed datasets and pooled model estimates from each of the three rule-based feature selection pipeline criteria using Rubin’s rules (mice::pool). Metabolites were considered candidates if they fulfilled the three specified criteria.

We next evaluated internal validity of the metabolites by bootstrapping (100 iterations) the complete rule-based feature selection pipeline whereby each resampled dataset maintained the distribution of classes (HCs, PSC pre-LT and PSC post-LT) from the original sample. Within each bootstrap sample, we generated 40 imputed datasets and pooled model estimates within each resampled dataset using Rubin’s rules. A metabolite that fulfilled all three criteria in a bootstrap sample was considered a winner within that sample. This enabled us to rank the metabolites by how frequently they appeared in the 100 bootstrap samples.

#### Time-to-event modelling (targeted, cross-sectional cohorts)

We used Kaplan–Meier survival curves to visualize the survival distributions of groups categorized by tertiles of circulating ImP levels. For analyses of rPSC, longitudinal ImP measurements from patients who had undergone LT once for PSC but with no recorded diagnosis of rPSC at the time of sampling were modelled as time-dependent covariates using Cox proportional hazards regression with start–stop intervals.

#### Longitudinal analyses

To test whether ImP overall changed longitudinally in the longitudinal cohort, we fitted a mixed-effects model using nmle::gls with ImP (log_2_) as outcome and time (continuous), age at LT and sex as fixed covariates. We defined an autoregressive correlation structure that allowed for subject-specific intercepts and slopes while allowing correlation between two observations within the same subject to decrease as the time differences between them increased.

#### Experimental data analyses

All cell culture experiments were performed in ≥3 biological replicates. Unless specified otherwise, *P* < 0.05 was considered statistically significant. To assess whether the effects of treatment on various outcomes differed depending on genetic background, we included interaction terms between treatment type and genetic background in our analysis. For ordinal outcomes, we applied the aligned rank transform for nonparametric factorial ANOVAs, while for continuous outcomes, we used ANOVA. For ANOVA, we visually inspected the model’s residuals to detect any clear departures from normality. We used two-sided Mann–Whitney *U*-tests to test for differences in distributions of ordinal and continuous variables.

#### Other statistical analyses

We tested the strengths of bivariate linear relationships using Spearman’s rank correlation. Where indicated, the Benjamini–Hochberg FDR procedure was performed to account for multiple testing.

R was used for all data processing, visualization and statistical analyses.

### Reporting summary

Further information on research design is available in the Nature Portfolio Reporting Summary linked to this article.

## Supplementary information

Supplementary Information Supplementary Note and Supplementary Figs. 1–5

Reporting Summary

Supplementary Tables 1–16 Multi-tab spreadsheet. The first tab shows an overview of the panes, followed by 16 panes: one for each supplementary table

Supplementary Dataset 1 Source data for Supplementary Fig. 1

Supplementary Dataset 2 Source data for Supplementary Fig. 2

Supplementary Dataset 3 Source data for Supplementary Fig. 3b

Supplementary Dataset 4 Source data for Supplementary Fig. 3c

Supplementary Dataset 5 Source data for Supplementary Fig. 4

Supplementary Dataset 6 Source data for Supplementary Fig. 5

## Source data

Source Data Fig. 1 Statistical source data

Source Data Fig. 2 Statistical source data

Source Data Fig. 3 Statistical source data

Source Data Fig. 4 Statistical source data

Source Data Fig. 5 Statistical source data

Source Data Fig. 6 Statistical source data

Source Data Fig. 7 Statistical source data

Source Data Extended Data Fig. 1 Statistical source data

Source Data Extended Data Fig. 2 Statistical source data

Source Data Extended Data Fig. 3 Statistical source data

Source Data Extended Data Fig. 4 Statistical source data

Source Data Extended Data Fig. 5 Statistical source data

Source Data Extended Data Fig. 6 Statistical source data

Source Data Extended Data Fig. 7 Statistical source data

Source Data Extended Data Fig. 8 Statistical source data

Source Data Extended Data Fig. 9 Statistical source data

Source Data Extended Data Fig. 10 Statistical source data

## Data Availability

Individual participant data have not been deposited in public repositories owing to Norwegian regulations and lack of consent, but will be made available to other researchers upon reasonable request. A request should be e-mailed to the corresponding author, with a very brief description of the aim and methods of the planned analyses. If this is covered by participant consent, an amendment will be filed for ethical approval of the analyses, and a material and data transfer agreement must be established between the institutions. Source data underlying all figures are published alongside this article. Source data underlying supplementary figures are provided in the Supplementary Data. RNA sequencing data have been deposited to Gene Expression Omnibus (accession number GSE341409). Correspondence and requests for materials or data should be addressed to the corresponding author. Source data are provided with this paper.

## Code availability

The analysis RMarkdown files and associated code are available via Zenodo at https://doi.org/10.5281/zenodo.21531744 (ref. ^51^).
