# Supplementary material for: Gut microbiota-derived imidazole propionate promotes primary sclerosing cholangitis via p38 signalling
Source: Nat Metab. 2026 Sep 4;8(9):1910–25. doi: 10.1038/s42255-026-01600-1 (PMC13597309; doi:10.1038/s42255-026-01600-1)
Supplement: Supplementary file 1 [file 42255_2026_1600_MOESM1_ESM.pdf]

nature metabolism

Supplementary information

https://doi.org/10.1038/s42255-026-01600-1

Gut microbiota-derived imidazole propionate promotes primary sclerosing cholangitis via p38 signalling

In the format provided by the authors and unedited

SUPPLEMENTARY NOTE

Definitions in the post-transplant cohort

Recurrent PSC (rPSC) after liver transplantation was analyzed as a secondary outcome. rPSC was defined according to Graziadei *et al.*,[1] with either cholangiographic findings or histology compatible with PSC. People with complications related to the hepatic artery were excluded, here defined as arterial strictures necessitating intervention with interventional radiology or surgery. As a modification of Graziadei, people with ductopenic rejection, early bile duct strictures, isolated anastomotic strictures, or donor–recipient ABO incompatibility were not excluded.

All available protocol and clinically indicated liver biopsies were retrieved from routine pathology archives and re-evaluated individually by two pathologists experienced with hepatobiliary and transplant-related pathology. The pathologists were blinded for the participant identity and original diagnosis on the histology reports. The pathologists concluded with one main diagnosis and up to two additional diagnoses. Samples with discrepant conclusions on the number of portal tracts, criteria for rPSC, and final diagnoses were reviewed jointly by the two pathologists for consensus. Biopsies with fibrous cholangitis and/or fibro-obliterative lesions were diagnosed as rPSC.

All available magnetic resonance cholangiography protocols and clinically indicated magnetic resonance cholangiography examinations were also re-evaluated by two radiologists. The radiologists were blinded for the participant identity and original evaluation of the MRC examinations. The assessment of recurrent primary sclerosing cholangitis (rPSC) was categorized using the following criteria: 1) definitely present, 2) probably present, 3) equivocal, 4) probably absent, and 5) definitely absent. For the final analysis, categories 1 (definitely present) and 2 (probably present) were accepted as rPSC.

A diagnosis of rPSC was set at the first date of a biopsy or MRC diagnostic for rPSC as defined above, in the absences of arterial problems in the clinical history. When there was a disagreement between radiologists, the first date of a rPSC diagnosis was used given that there was consensus of rPSC based on a later MRC. In cases where there was no clear consensus later, and the biopsy was not diagnostic for rPSC, patient charts were examined for either a clinical diagnosis of rPSC or the occurrence of signs and symptoms of rPSC.

For analyses aiming to estimate the association between ImP and the hazard of rPSC, longitudinal post-transplant measurements taken during routine follow up assessments[2] were used*,* and follow-up was defined from the first available post-transplant sample until rPSC diagnosis or censoring at the last available liver biopsy and/or magnetic resonance cholangiography (MRC) re-evaluated for signs of rPSC. Individuals who died without rPSC were censored at their last such biopsy/MRC. To avoid reverse causation, ImP measurements obtained within 90 days prior to the

recorded rPSC diagnosis were excluded from these analyses since disease onset likely preceded the formal diagnosis.

Untargeted metabolomics using ultra-high performance liquid chromatography-tandem mass spectroscopy (UPLC–MS/MS, Metabolon inc.)

In serum samples proteins were precipitated with methanol with vigorous shaking for 2 minutes and centrifugation, and the resulting extract was divided into fractions for two separate reverse phase (RP) methods with positive ion mode electrospray ionization (ESI), one for RP with negative mode ESI, and one for HILIC/UPLC–MS/MS with negative mode ESI. The organic solvents were evaporated and stored under nitrogen. The samples were reconstituted in solvents compatible with the ESI methods. The sample order for UPLC–MS/MS analysis was randomized. A pool of all analyzed samples was used as a technical replicate at different time points during the UPLC–MS/MS procedure, and a cocktail of quality control standards were spiked into every sample, collectively to monitor performance and to achieve chromatographic alignment.

UPLC–MS/MS was performed on a Waters ACQUITY and Thermo Scientific Q-Exactive high resolution/accurate mass spectrometer interfaced with a heated electrospray ionization (HESI-II) source and Orbitrap mass analyzer operated at 35,000 mass resolution. For the positive mode ESI, one aliquot was gradient-eluted from a C18 column (Waters UPLC BEH C18-2.1x100 mm, 1.7 µm) using water and methanol supplied with 0.05% perfluoropentanoic acid and 0.1% formic acid. The other aliquot was eluted from the same C18 column, but with acetonitrile, water, 0.05% perfluoropentanoic acid and 0.01% formic acid to better separate hydrophobic metabolites. For the negative mode ESI, one aliquot was gradient-eluted from another, but identical, C18 column using water, 6.5mM ammonium bicarbonate (pH 8), and the other was negatively ionized after elution from a HILIC column (Waters UPLC BEH Amide 2.1x150 mm, 1.7 µm) using a gradient of water and acetonitrile with 10mM ammonium formate (pH 10.8). Another aliquot was analyzed using basic negative ion optimized conditions using a separate dedicated C18 column. The basic extracts were gradient eluted from the column using methanol and water, however with 6.5mM ammonium bicarbonate at pH 8. The fourth aliquot was analyzed via negative ionization following elution from a HILIC column (Waters UPLC BEH Amide 2.1x150 mm, 1.7 µm) using a gradient consisting of water and acetonitrile with 10mM ammonium formate, pH 10.8. The MS analysis alternated between MS and data-dependent MS^n scans using dynamic exclusion. The scan range varied slightly between methods but covered 70-1000 m/z. Raw data was extracted, and peaks were identified, and quality-control processed. Peak quantification was performed using area-under-the-curve. Block correction using median normalization was performed to normalize for day-to-day instrument variability. Peak abundance data were normalized

by raw area counts ("OrigScale"), imputed (see *handling of missing data*), log_2-transformed and scale-normalized with centering.

Targeted ImP and urocanate measurements

ImP and urocanate in serum samples were measured using targeted UPLC–MS/MS. Briefly, 25 µL of serum samples were extracted using 6 volumes of acetonitrile containing 100 nM of internal standards (ImP-13C3 and urocanate-13C3, Astra Zeneca, Cambridge, UK) before drying the samples under a flow of nitrogen. Then, the samples were reconstituted with 5% HCl (37%) in 1-butanol, subjected to n-butyl ester derivatization and finally reconstituted in 150 µL water:acetonitrile (9:1). ImP and urocanate levels were determined by UHPLC–MS/MS analysis using multiple reaction monitoring of the transitions 197/81 and 195/93 respectively.

Liver histology and immunohistochemistry

Liver specimens were fixed in 10% buffered formalin and embedded in paraffin, and 3-5 µm sections were obtained and processed for hematoxylin and eosin (H&E) and Sirius Red/Fast Green (SR/FG) according to standard protocols. For immunohistochemistry, endogenous peroxidase activity was blocked by a 30-minute incubation in methanolic hydrogen peroxide (2.5%). Antigens were retrieved, as indicated by the vendor, by applying Proteinase K (Dako, Glostrup, Denmark, code S3020) for 10 minutes at room temperature. Sections were then incubated overnight at 4°C with primary antibodies (CK19: abcam, code #ab133496, dilution 1:100; p-mTOR: Cell Signaling Technologies, code #2976S, dilution 1:100; CD3: Dako, Agilent, M725401, dilution 1:100; CD20: Dako, Agilent, M0755, dilution 1:100; CD68: Dako, Agilent. Code: M0876, dilution 1:100). Then, samples were rinsed twice with phosphate buffered saline (PBS) for 5 minutes, incubated for 45 minutes at room temperature with Dako REAL EnVision Detection System (codes K4003 and K5007, Dako, Agilent Technologies, Glostrup, Denmark), Diaminobenzidine (code: K3468, Dako, Agilent Technologies, Glostrup, Denmark) was used as substrate, and sections were counterstained with hematoxylin. Sections were examined in a coded fashion by Leica Microsystems DM4500B Light Microscopy, equipped with Leica K3C Videocam (Weltzlar, Germany) and LAS X Core software. All stained slides were scanned by a digital scanner (Aperio Scanscope CS System, Aperio Digital Pathology, Leica Biosystems, Milan, Italy) and processed by ImageScope. Portal inflammation was assessed on H&E stains. Fibrosis was evaluated on SR/FG- stained slides. Portal inflammation was scored as following: 0=no inflammation; 1=minimal inflammation; 2=greater than minimal inflammation in less than one third of portal tracts; 3=greater than minimal inflammation in one third to two thirds of portal tracts; 4=greater than minimal inflammation in more than two thirds of portal tracts. Fibrosis was scored as following: 0=no fibrosis; 1=portal fibrosis; 2=portal fibrosis with periportal fibrosis; 3= bridging fibrosis; 4=cirrhosis. For liver fibrosis further quantification, SR stained slides were processed by Aperio eSlide Manager (v12.4.0.5043) and HALO® and HALO AI ® (v4.1; Indica Labs, Albuquerque, NM, USA) softwares. AI-based MiniNet network

was trained to classify portal tracts within the liver in human samples and in murine samples, respectively. The extent of SR stain was assessed both on total liver area and on AI-classified portal tracts by using positive area quantification algorithm and is expressed as percentage of portal tract area.

The extent of DR was evaluated by CK19 immunoreactivity. The area occupied by CK19-positive cells was quantified by an image analysis algorithm on ImageScope, and DR extent was expressed as the percentage of the parenchymal area occupied by reactive ductules.[3]

For p-mTOR expression, the number of positive biliary epithelial cells was automatically calculated by an algorithm on the entire section, and then a SQ scoring system was applied (0 ≤ 1%; 1=1-10%; 2=11-30%; 3=31-50%; 4 ≥ 51%).[4] All histological analysis were performed by two (GC and DO) independent experienced pathologists blindly both for animal and human liver specimens.

In vitro experiments

H69 were kindly gifted from Prof. Domenico Alvaro (Department of Translational and Precision Medicine of Sapienza University of Rome, Italy) and were cultured as previously described.[5] Before stimulation with ImP, urocanate or vehicle (1 % DMSO), cells were growth in FBS-free culture media overnight and were then amino acid-deprived by replacing the medium with EBSS for 1h. Next, the cells were cultured for another four hours with ImP or urocanate at concentrations of 10 or 100 µM (Santa Cruz Biotechnology sc294276; CAS 1074-59-5 or sc214246; CAS 104-89-3) in EBSS-medium (Thermo Fischer Scientific). The experiment was performed in three independent biological replicates, each with two technical replicates. After stimulation, we harvested the cells, extracted RNA with RNeasy Mini Kit (Qiagen 74106) and measured RNA concentration and quality using Nanodrop.

RNA sequencing and data analysis

After quality check, RNA sequencing was performed by Biomarker Technologies (BMK) GmbH. Then cDNA libraries were constructed using ≥500 ng RNA per sample (VAHTS Universal V6 RNA-seq Library Prep Kit Illumina NR604-02), following manufacturer’s recommendations. The effective concentration of the library was determined to be at least 2nM. The resultant sequencing libraries were then submitted for sequencing on the Illumina NoveSeq X platform, PE150 mode.

Counts data were imported and genes with a sum of counts across all conditions of less than ten were removed. A DESeqDataSet object was made of the count data and metadata and differential expression analyses were performed using DESeq2 using the relevant contrasts (designs). For the principal component analysis (PCA) no contrast was specified, and the *DESeq2::vst* function was used to normalize the data prior performing the PCA.

Gene set enrichment analyses were performed using the *fgsea::fgsea* function in R. Genes were ranked by the t-statistic from the DESeq2 analyses. The HALLMARK (h.all.v2023.1.Hs.symbols.gmt) and GO biological processes (c5.go.bp.v2023.1.Hs.symbols.gmt) pathways (lists of gene sets) were used with a minimum size of five for each gene set.

Ex vivo experiments

Cholangiocytes were collected from the bile ducts of individuals with PSC undergoing ERCP at Oslo University Hospital (Oslo, Norway) as described in [6–8]. All donors had given written informed consent prior to ERCP, and the use of cells from human livers and bile ducts was approved by the Regional Ethics Committee (2012-286/2016-1540). Organoids were given fresh culture medium every 48 hours and were passaged every 5–7 days depending on their quality and quantity. Established organoids were treated for six days with ImP (Santa Cruz sc294276), urocanate (Santa Cruz sc214246) or vehicle (DMSO 1%) dissolved in the medium.

A titration with 10, 50 and 100 µM ImP on one organoid model indicated that 50 µM ImP was required to induce a robust increase in the secretion of pro-inflammatory and pro-fibrotic markers in the culture medium. At 100 µM the cells showed overt signs of toxicity. Hence, 50 µM was chosen for all subsequent experiments. Supernatants were collected every 48 hours and stored at -20°C until further analysis.

Multiplex analysis

Supernatants from patient-derived cholangiocyte organoids were thawed on ice, diluted 2-fold in Calibrator Diluent RD6-52, and TNF-α, ICAM1,CXCL2, IFN-γ, IL-6, IL-1β, FAP, MMP-9, and Fibronectin were measured using a human magnetic Luminex® assay (R&D Systems/Bio-Techne) according to the manufacturer's instuctions. Concentrations of selected analytes were estimated using a Bio-Plex 200 system (Bio-Rad) with Bio-Plex ManagerTM Software Version 6.2. Values below or above the limits of detection were imputed to the minimum or maximum, respectively.

Supplementary references

1. Graziadei, I. W. *et al.* Recurrence of primary sclerosing cholangitis following liver transplantation. *Hepatology* **29** 1050–1056 (1999) doi:10.1002/hep.510290427.
2. Braadland, P. R. *et al.* Clinical and biochemical impact of vitamin B6 deficiency in primary sclerosing cholangitis before and after liver transplantation. *J. Hepatol.* **79** 955–966 (2023) doi:10.1016/j.jhep.2023.05.038.

3. Overi, D. *et al.* Role of ductular reaction and ductular–canalicular junctions in identifying severe primary biliary cholangitis. *JHEP Rep.* **4** 100556 (2022) doi:10.1016/j.jhepr.2022.100556.
4. de Jong, I. E. M. *et al.* Persistent biliary hypoxia and lack of regeneration are key mechanisms in the pathogenesis of posttransplant nonanastomotic strictures. *Hepatology* **75** 814–830 (2022) doi:10.1002/hep.32166.
5. Carpino, G. *et al.* Neoplastic Transformation of the Peribiliary Stem Cell Niche in Cholangiocarcinoma Arisen in Primary Sclerosing Cholangitis. *Hepatology* **69** 622–638 (2019) doi:10.1002/hep.30210.
6. Frank, A. K. *et al.* Single-Cell Transcriptomic Profiling of Cholangiocyte Organoids Derived from Bile Ducts of Primary Sclerosing Cholangitis Patients. *Dig. Dis. Sci.* **69** 3810–3823 (2024) doi:10.1007/s10620-024-08570-y.
7. Tysoe, O. C. *et al.* Isolation and propagation of primary human cholangiocyte organoids for the generation of bioengineered biliary tissue. *Nat. Protoc.* **14** 1884–1925 (2019) doi:10.1038/s41596-019-0168-0.
8. Sampaziotis, F., de Brito, M. C., Geti, I., Bertero, A., Hannan, N. R. & Vallier, L. Directed differentiation of human induced pluripotent stem cells into functional cholangiocyte-like cells. *Nat. Protoc.* **12** 814–827 (2017) doi:10.1038/nprot.2017.011.

SUPPLEMENTARY FIGURES 1-5

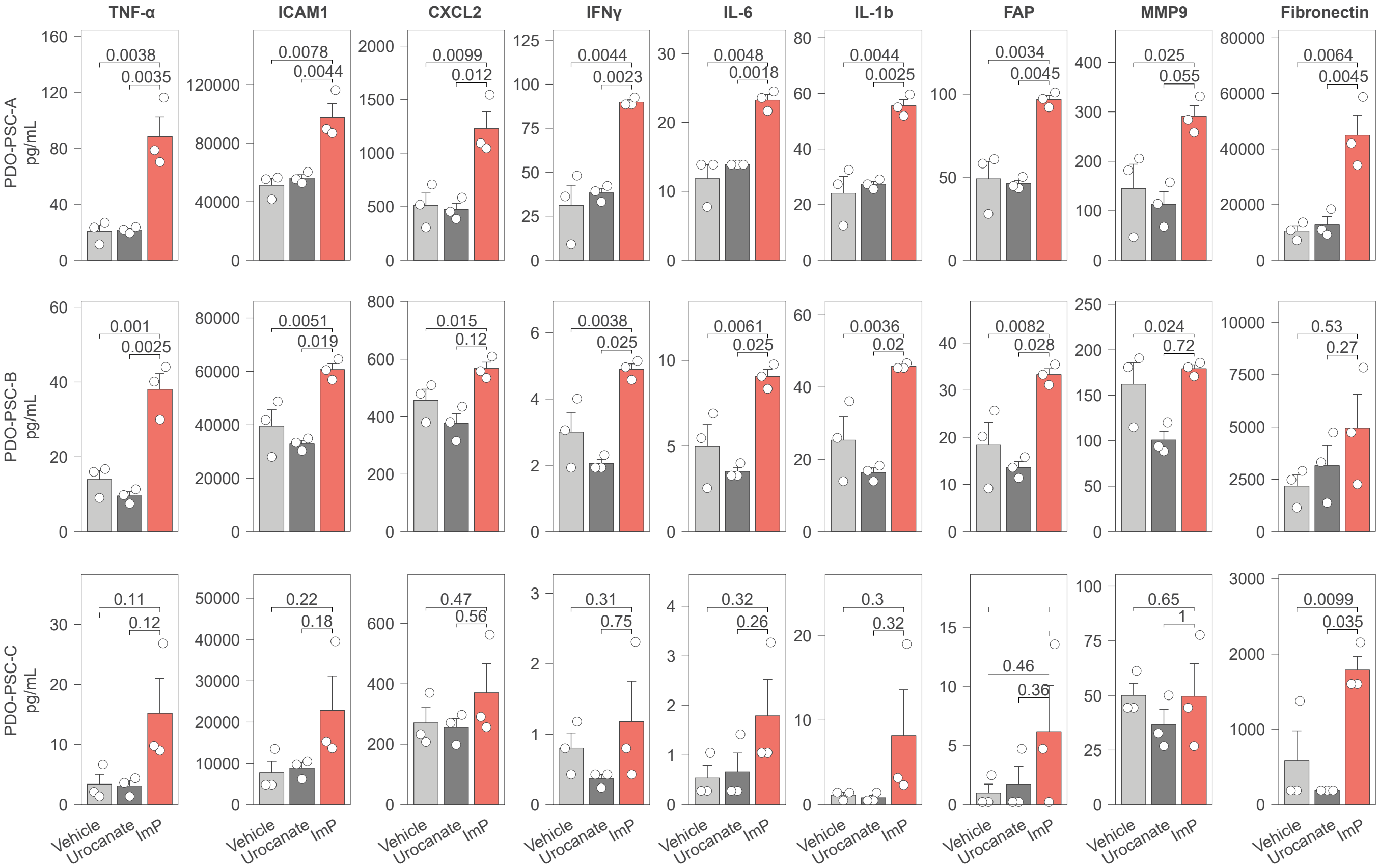

Supplementary Fig. 1. **Cytokine levels in supernatants of patient-derived cholangiocyte organoids treated with imidazole propionate**. Concentrations of pro-inflammatory (TNFα, ICAM1, CXCL2, INFγ, IL-6, IL-1b) and pro-fibrotic (FAP, MMP9, Fibronectin) factors in the media of cholangiocyte organoids derived from three independent donors with PSC (arbitrarily named Patients Derived cholangiocyte Organoids (PDO)-PSC-A treated with 50 µM imidazole propionate (ImP), 50 µM urocanate (Uro) or vehicle for 4 days. Data are presented as absolute concentrations. Values outside the limits of detection were imputed to the minimum or maximum. Each of the three organoid models were run in technical triplicates. Bars represent the mean, and error bars represent the standard error of the mean (SEM). Statistical significance was calculated using ANOVA with Tukey's honest significant difference post-hoc test. CXCL2: C-X-C motif ligand 2, FAP: Fibroblast Activation Protein, ICAM1: Intercellular Adhesion Molecule 1, IFNγ: Interferon gamma, IL-1b: Interleukin 1 beta, IL-6: Interleukin 6, MMP9: Matrix MetalloProteinase-9, PDO: patient-derived organoid, TNF-α: Tumor Necrosis Factor alpha.

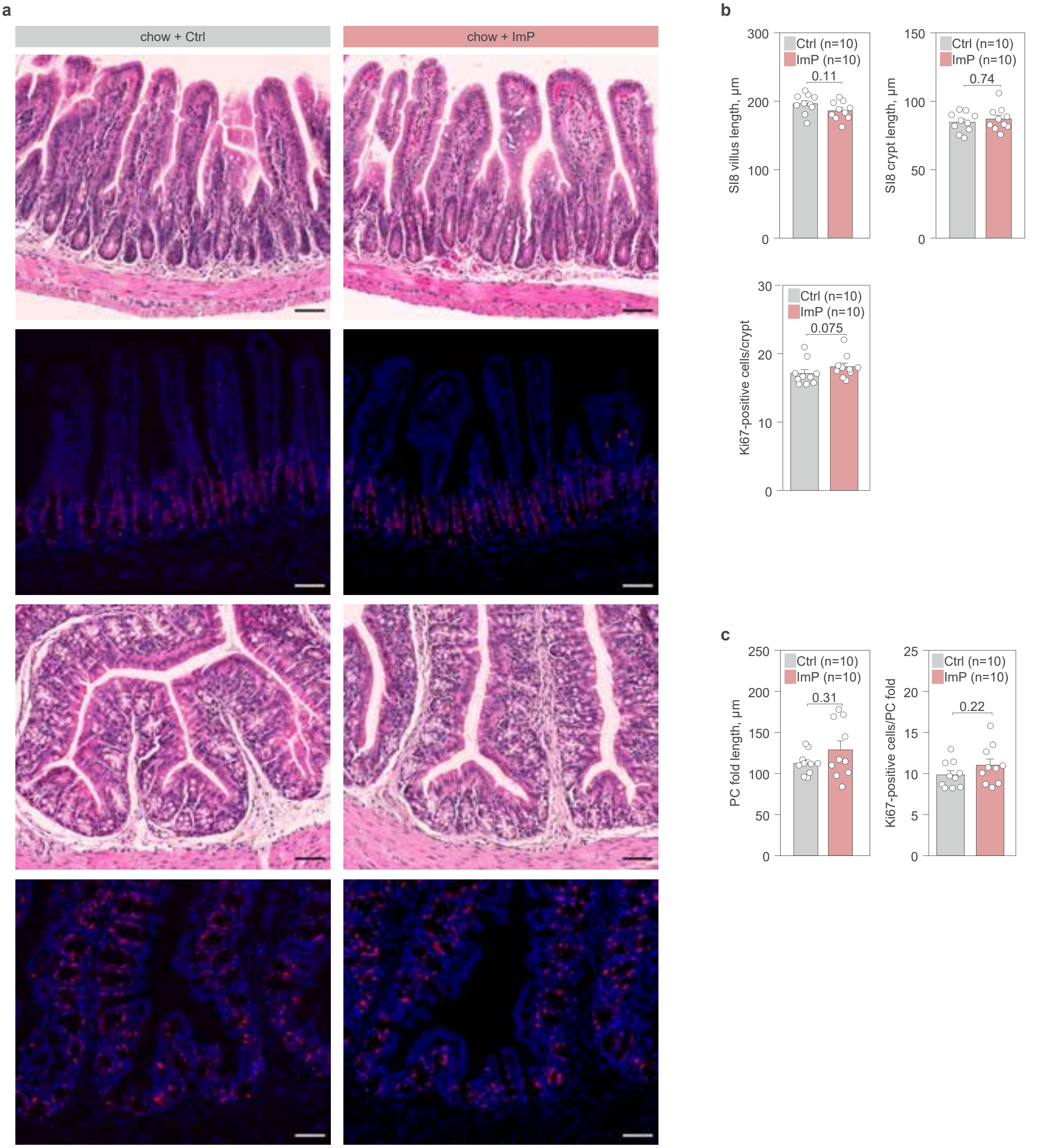

Supplementary Fig. 2. Small and large intestine crypt and villi anatomy in wild type mice treated with and without imidazole propionate. a-b. Representative pictures and relative quantification for villi and crypt structure and immune staining for Ki67 in small intestinal terminal ileum (Si8) and (**c**) in the proximal colon from wild type mice used for main Fig. 7. Scale bars = 50 µm. Bars represent the mean, and error bars represent the standard error of the mean (SEM). P-values were calculated using two-sided Mann-Whitney U tests. N = 10 per group.

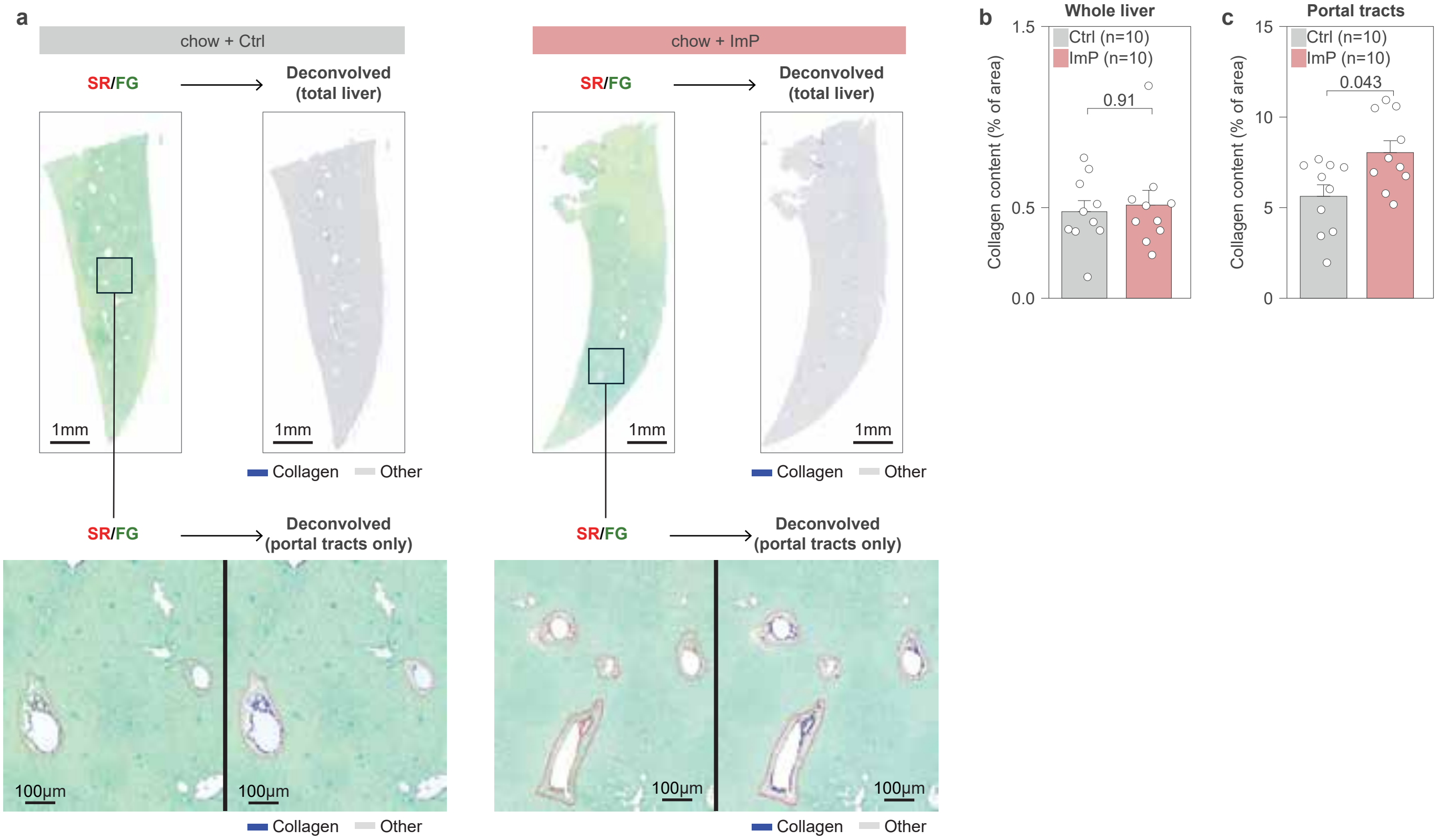

Supplementary Fig. 3. Artificial intelligence-based evaluation of liver fibrosis in wild type mice treated with and without imidazole propionate. a-c. Representative pictures and relative quantification of collagen content area in the mouse (b) whole livers and (c) portal areas (N = 10 per group, same mice used for data presented in main Fig. 7). Bars represent the mean, and error bars represent the standard error of the mean (SEM). P-values were calculated using two-sided Mann-Whitney U tests.

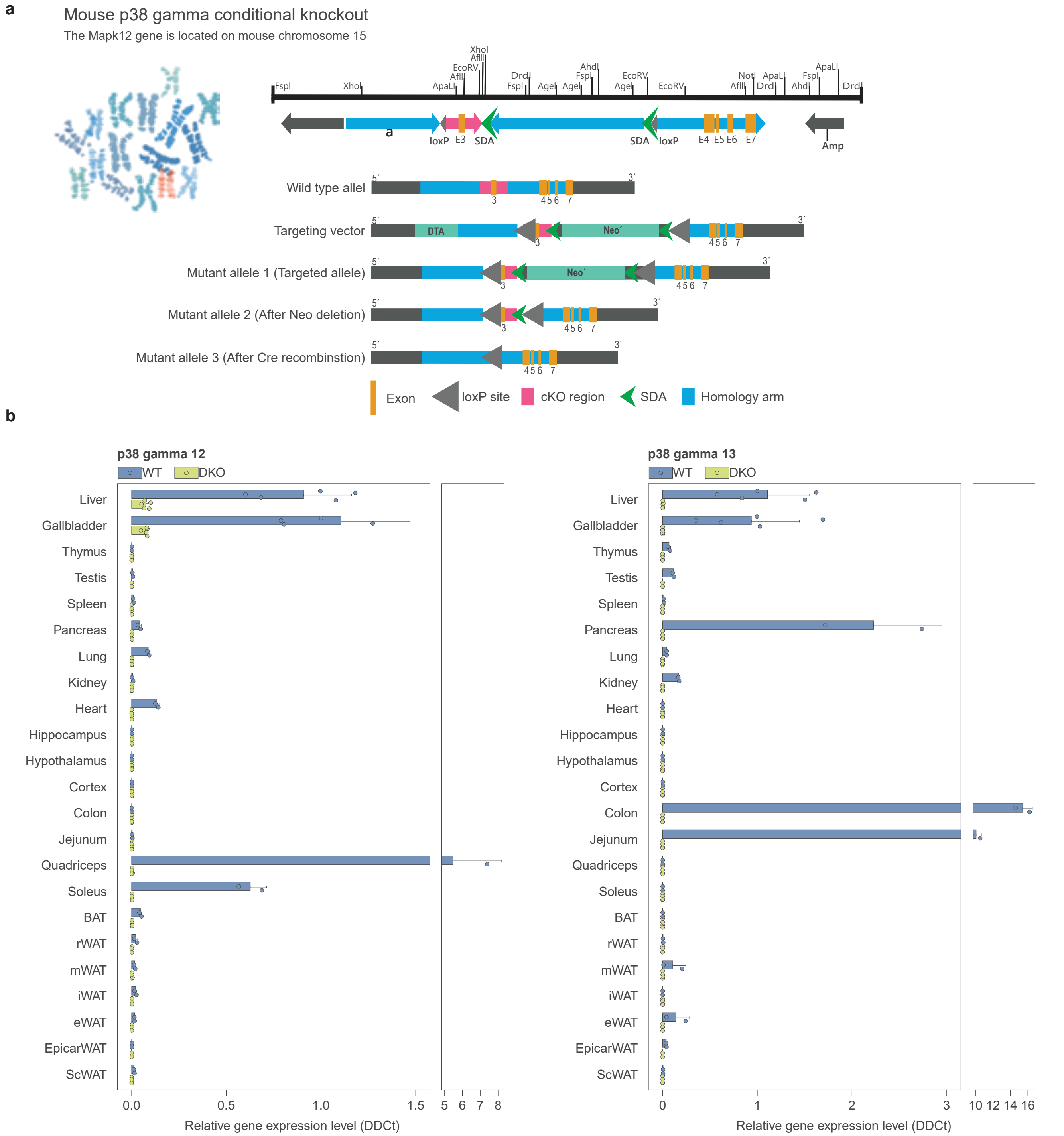

Supplementary Fig. 4. Generation of p38γ-δ knock out mice (DKO). a. Schematic representation of the p38γ-δ (Mapk12-13) gene-targeting strategy. **b**. Relative expression of p38γ-δ gene in different tissues in DKO and WT. n = 2 WT and 5 DKO mice used. Data are plotted as mean+standard deviation. BAT: brown adipose tissue, rWAT: peri-renal white adipose tissue, mWAT: mesenterial white adipose tissue, iWAT: inguinal white adipose tissue, eWAT: epididymal white adipose tissue, EpicarWAT: epicardial white adipose tissue, ScWAT: sub-cutaneous white adipose tissue.

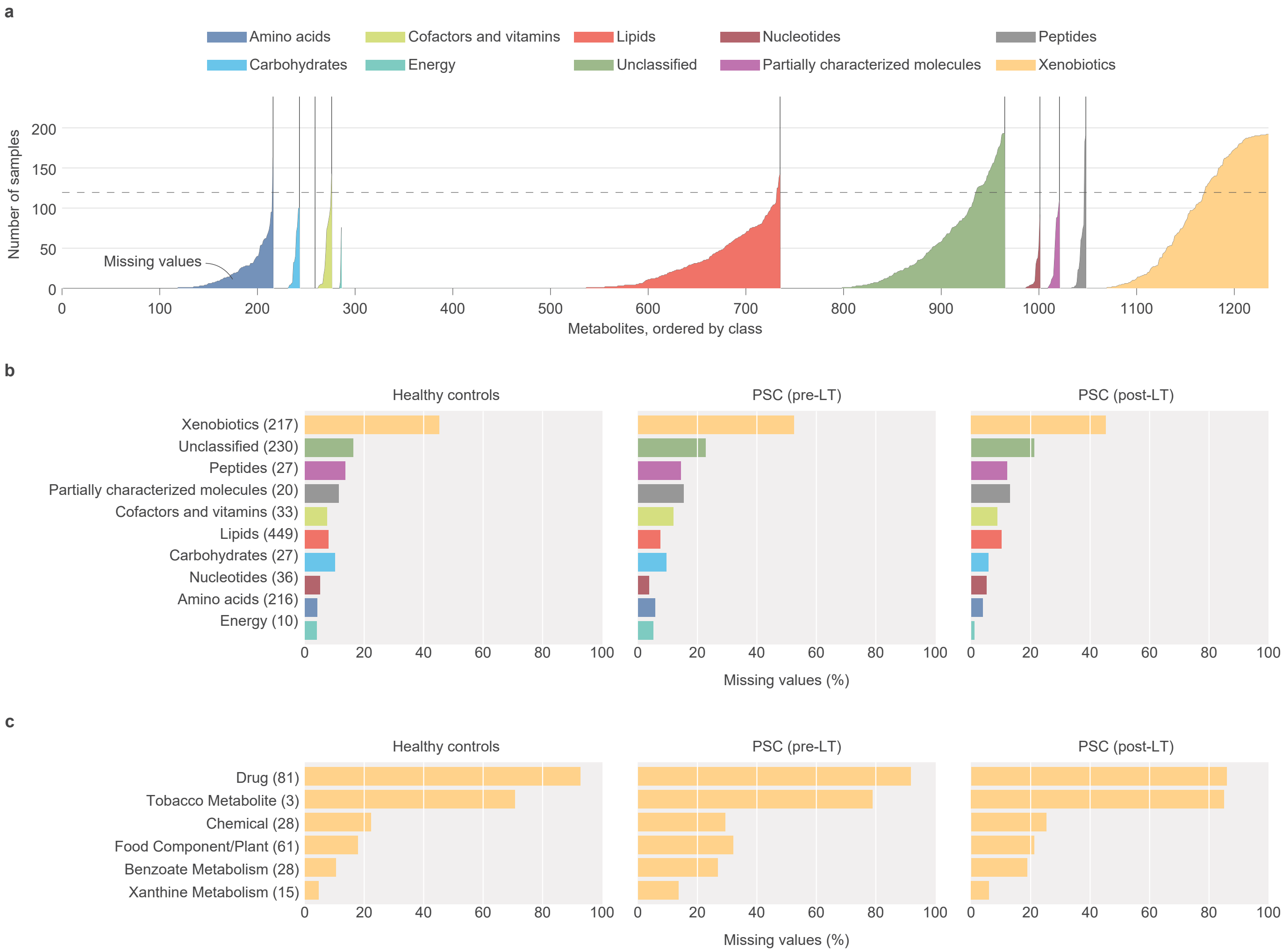

Supplementary Fig. 5. Visualization of missing data in the untargeted metabolomic profiling (discovery) cohort. a. The filled areas indicate missing metabolites, and the horizontal, dashed line indicate the 50% detection threshold used to exclude metabolites from further analyses. Metabolites were sorted ascendingly by the number of metabolites missing within each class, and the y-axis indicate the number of samples (including healthy controls, PSC pre-LT and PSC five years post-LT without a recurrent PSC diagnosis). **b**. Shows, for each group, the percentage of missing values of each metabolite pathway ('super pathway'). The annotated numbers indicate the number of metabolites detected for each 'super pathway'. **c**. Shows the fraction of missing values for sub-pathways within the xenobiotics 'super pathway'. The high degree of missingness for e.g. drugs and tobacco metabolites provides a good rationale for selectively imputing missing xenobiotic metabolite levels to zero. HC: Healthy control, LT: Liver transplantation, PSC: Primary sclerosing cholangitis.
