# Supplementary material for: Gut microbiota-derived imidazole propionate promotes primary sclerosing cholangitis via p38 signalling
Source: Nat Metab. 2026 Sep 4;8(9):1910–25. doi: 10.1038/s42255-026-01600-1 (PMC13597309; doi:10.1038/s42255-026-01600-1)
Supplement: Supplementary file 2 [file 42255_2026_1600_MOESM2_ESM.pdf]

nature portfolio

Corresponding author(s): Johannes Roksund Hov

Last updated by author(s): Jul 26, 2026

Reporting Summary

Nature Portfolio wishes to improve the reproducibility of the work that we publish. This form provides structure for consistency and transparency in reporting. For further information on Nature Portfolio policies, see our Editorial Policies and the Editorial Policy Checklist.

Statistics

For all statistical analyses, confirm that the following items are present in the figure legend, table legend, main text, or Methods section.

n/a	Confirmed	
☐	☒	The exact sample size (n) for each experimental group/condition, given as a discrete number and unit of measurement
☐	☒	A statement on whether measurements were taken from distinct samples or whether the same sample was measured repeatedly
☐	☒	The statistical test(s) used AND whether they are one- or two-sided<br>*Only common tests should be described solely by name; describe more complex techniques in the Methods section.*
☐	☒	A description of all covariates tested
☐	☒	A description of any assumptions or corrections, such as tests of normality and adjustment for multiple comparisons
☐	☒	A full description of the statistical parameters including central tendency (e.g. means) or other basic estimates (e.g. regression coefficient) AND variation (e.g. standard deviation) or associated estimates of uncertainty (e.g. confidence intervals)
☐	☒	For null hypothesis testing, the test statistic (e.g. F, t, r) with confidence intervals, effect sizes, degrees of freedom and P value noted<br>*Give P values as exact values whenever suitable.*
☒	☐	For Bayesian analysis, information on the choice of priors and Markov chain Monte Carlo settings
☐	☒	For hierarchical and complex designs, identification of the appropriate level for tests and full reporting of outcomes
☐	☒	Estimates of effect sizes (e.g. Cohen's d, Pearson's r), indicating how they were calculated

Our web collection on statistics for biologists contains articles on many of the points above.

Software and code

Policy information about availability of computer code

Data collection	Internal database system at Oslo University Hospital (Medinsight)
Data analysis	R was used for all data processing, visualization, and statistical analyses, HALO® and HALO AI ®, LAS X Core, Bio-Plex ManagerTM for in vivo and ex vivo data analysis

For manuscripts utilizing custom algorithms or software that are central to the research but not yet described in published literature, software must be made available to editors and reviewers. We strongly encourage code deposition in a community repository (e.g. GitHub). See the Nature Portfolio guidelines for submitting code & software for further information.

Data

Policy information about availability of data

All manuscripts must include a data availability statement. This statement should provide the following information, where applicable:

- Accession codes, unique identifiers, or web links for publicly available datasets
- A description of any restrictions on data availability
- For clinical datasets or third party data, please ensure that the statement adheres to our policy

Individual participant data have not been deposited in public repositories due to Norwegian regulations and lack of consent, Individual participant data will be made available to other researchers upon reasonable request, following amendments to the ethical approvals and a material and data transfer agreement between the institutions. Likely time-frame: 3 months. However, fully anonymized source data for all figures will be provided with this paper.

Research involving human participants, their data, or biological material

Policy information about studies with human participants or human data. See also policy information about sex, gender (identity/presentation), and sexual orientation and race, ethnicity and racism.

Reporting on sex and gender	Sex is reported in all the human data presented. Participant sex is based on the population registry and hence not self-reported. The initial comparisons of key features (ImP level) between patients and controls showed no effect of sex (Extended data figure 2D), hence sex was not studied specifically later. Additional sex-focused data must be considered clearly post-hoc and would have very limited high scientific value. We also lack permission and consent to deposit individual level data. Taken together, data disaggregated for sex is therefore not provided for source data nor for summary tables or individual comparisons/statistical tests. The underlying individual dat of the study may be shared according to the data availability statement.
Reporting on race, ethnicity, or other socially relevant groupings	Ethnicity is not considered relevant or reported in our data analysis
Population characteristics	Patients with PSC, PBC, MASLD, ALD and IBD under follow-up in specialized center for the treatment and follow-up for chronic liver diseases.
Recruitment	Performed at local centers or at a tertiary care hospital
Ethics oversight	No specific risk for the patients as this is a cross-sectional observational study performed in already collected blood samples.

Note that full information on the approval of the study protocol must also be provided in the manuscript.

Field-specific reporting

Please select the one below that is the best fit for your research. If you are not sure, read the appropriate sections before making your selection.

☒ Life sciences ☐ Behavioural & social sciences ☐ Ecological, evolutionary & environmental sciences

For a reference copy of the document with all sections, see nature.com/documents/nr-reporting-summary-flat.pdf

Life sciences study design

All studies must disclose on these points even when the disclosure is negative.

Sample size	5-10 mice per group in each experiment, 5 cells replicate per condition, 3 organoids separate experiments generated from 3 independent donator
Data exclusions	None of mice have been excluded from the analysis
Replication	No applicable
Randomization	Mice were randomly assigned to IMP or Vehicle (1:1 per cage)
Blinding	All histological quantification were performed blindly by two expert pathologists and MRIs re-reviewed blinded by two radiologists

Reporting for specific materials, systems and methods

We require information from authors about some types of materials, experimental systems and methods used in many studies. Here, indicate whether each material, system or method listed is relevant to your study. If you are not sure if a list item applies to your research, read the appropriate section before selecting a response.

Materials & experimental systems

n/a	Involved in the study	
☐	☒	Antibodies
☐	☒	Eukaryotic cell lines
☒	☐	Palaeontology and archaeology
☐	☒	Animals and other organisms
☐	☒	Clinical data
☒	☐	Dual use research of concern
☒	☐	Plants

Methods

n/a	Involved in the study	
☒	☐	ChIP-seq
☒	☐	Flow cytometry
☒	☐	MRI-based neuroimaging

Antibodies

Antibodies used	CK19: abcam, code #ab133496, dilution 1:100; p-mTOR: Cell Signaling Technologies, code #2976S, dilution 1:100; CD3: Dako, Agilent, M725401, dilution 1:100; CD20: Dako, Agilent, M0755, dilution 1:100; CD68: Dako, Agilent. Code: M0876, dilution 1:100
Validation	Not performed due to commercially available antibodies used in all the experiments

Eukaryotic cell lines

Policy information about cell lines and Sex and Gender in Research

Cell line source(s)	H69 were kindly gifted from Prof. Domenico Alvaro, organoids were derived from patient bile duct brushes
Authentication	Not performed
Mycoplasma contamination	Tested negative (several times)
Commonly misidentified lines (See ICLAC register)	Not used

Animals and other research organisms

Policy information about studies involving animals; ARRIVE guidelines recommended for reporting animal research, and Sex and Gender in Research

Laboratory animals	C57b6/J mice and Swiss Webster germ free mice, Gt(ROSA)26Sortm16(cre)Arte – Cre Deleter were purchased from Charles River, MAPK12-13 flox mice were generated by Charles River upon investigator request
Wild animals	not used
Reporting on sex	only male mice were used. The main reason for this is the 3Rs (reduce, refine replace) where sex differences would necessitate a doubling of the number of animals. Male mice were chosen as PSC is a male dominated disease (2:1 male:female ratio)
Field-collected samples	not used
Ethics oversight	Dnr 5.8.18-16056/2019 approved by the Gothenburg animal ethics committee

Note that full information on the approval of the study protocol must also be provided in the manuscript.

Clinical data

Policy information about clinical studies

All manuscripts should comply with the ICMJE guidelines for publication of clinical research and a completed CONSORT checklist must be included with all submissions.

Clinical trial registration	(No clinical interventions performed), but the research projects are covered by the follwoing approvals: Norway (2010/1540, 2011/2572, 2015/2140, no.568878, 2012/286, 2016/1690, 568878), Sweden Dnr: 571-18 and 2018/1111-32, Italy PBC322 (n.2969), n.5129 (541/1). USA PSC Mayo Clinic Institutional Review Board (IRB) #s 670-02 and 16-005892. Denmark PBC ethical committee of the Central Denmark Region (1-10-72-146-16)
Study protocol	Blood/stool sampling of patients with PBC, PSC, MASLD, ALD, IBD and healthy controls at baseline and/or during follow-up and after transplantation. No specific interventions have been performed in all the cohorts analyzed
Data collection	Clinical data collected at time of blood/stool sampling.
Outcomes	Investigated clinical (observational outcomes): 1. Transplant-free survival and 2. recurrence of PSC post-transplantation

Plants

Seed stocks	not used
Novel plant genotypes	not used
Authentication	not applicable
